# Brucein D, a Naturally Occurring Tetracyclic Triterpene Quassinoid, Induces Apoptosis in Pancreatic Cancer through ROS-Associated PI3K/Akt Signaling Pathway

**DOI:** 10.3389/fphar.2017.00936

**Published:** 2017-12-22

**Authors:** Zheng-Quan Lai, Siu-Po Ip, Hui-Jun Liao, Zheng Lu, Jian-Hui Xie, Zi-Ren Su, Yun-Long Chen, Yan-Fang Xian, Po-Sing Leung, Zhi-Xiu Lin

**Affiliations:** ^1^School of Chinese Medicine, Faculty of Medicine, The Chinese University of Hong Kong, Shatin, Hong Kong; ^2^Department of Clinical Pharmacy and Pharmaceutical Services, Shenzhen Sixth People’s Hospital – Nanshan Hospital, Shenzhen, China; ^3^Liver Cirrhosis Diagnosis and Treatment Center, Beijing 302 Hospital, Beijing, China; ^4^Guangdong Provincial Key Laboratory of Clinical Research on Traditional Chinese Medicine Syndrome, The Second Affiliated Hospital, Guangzhou University of Chinese Medicine, Guangzhou, China; ^5^Guangdong Provincial Key Laboratory of New Drug Development and Research of Chinese Medicine, Mathematical Engineering Academy of Chinese Medicine, Guangzhou University of Chinese Medicine, Guangzhou, China; ^6^School of Biomedical Sciences, Faculty of Medicine, The Chinese University of Hong Kong, Shatin, Hong Kong

**Keywords:** pancreatic cancer, brucein D, apoptosis, PI3K/Akt, ROS

## Abstract

Brucein D (BD), a major active quassinoid in *Brucea javanica*, has exhibited pronounced anticancer activities. However, the biologic mechanisms have not been fully explored. In this study, BD exhibited more potent cytotoxic effect on pancreatic cancer (PanCa) cell lines, while exerted weaker cytotoxic effects on GES-1 cells (non-tumorigenic). BD was shown to elicit apoptosis through inducing both the intrinsic and extrinsic mitochondria-mediated caspase activations. Furthermore, the BD-induced apoptotic effects were dependent on the accumulated reactive oxygen species (ROS) and inactivation of PI3K/Akt signaling pathway. Pretreatment with tempol completely prevented the cellular apoptosis induced by BD, and recovered the inactivation of AKT, which suggested ROS essentially involved in BD-elicited apoptosis and down-regulation of PI3K/Akt pathway. In addition, the results obtained from orthotopic xenograft in nude mice were congruent with those of the *in vitro* investigations. These results support the notion that BD held good potential to be further developed into an effective pharmaceutical agent for the treatment of PanCa.

## Introduction

Pancreatic cancer (PanCa) is an aggressive malignant disease usually diagnosed at an advanced stage. PanCa is at present the fourth dominating cause of cancer-oriented death in the Western word. In 2016, 53,070 new cases of PanCa were expected in the United States with 41,780 deaths (Siegel et al., 2016). Albeit advancement in diagnosis, radiotherapy, chemotherapy, and surgery, median life expectancy of PanCa patients post diagnosis is less than half a year and the overall 5-year survival rate is between 3 and 5% (Iovanna et al., 2012). Surgical operation is still the potential effective regime for management of this disease. However, absence of successful early diagnosis limits the surgical resection to merely about 10% of the patients. Therefore, most of PanCa patients will turn to chemotherapy and radiotherapy (Awasthi et al., 2011). However, these treatments may lead to various side effects. Moreover, resistance to chemotherapy is frequent and is intimately related to poor clinical outcomes. Gemcitabine (GEM) is the current first-line chemotherapeutic drug for PanCa, but the successful rate remains challenging (Kagawa et al., 2012). To reduce the PanCa mortality, it is vital to explore novel effective anti-PanCa agents with more favorable safety profile.

Apoptosis is among the most common mechanisms for counteracting cancer cell or sensitizing cells to the chemotherapeutic agents and radiation therapy (Ghobrial et al., 2005). It can be initiated through either extrinsic or intrinsic pathway (Denicourt and Dowdy, 2004). Extrinsic pathway is activated by specific factors like Fas-ligand, TNF-α, and TRAIL that activate caspase-8 and subquentcaspase-3 (Li et al., 1998). Another is the intrinsic pathway (mitochondrial), which is provoked by the translocation of cytochrome c across mitochondria (Arnoult et al., 2002). The released cytochrome c activates caspase cascades, eventually leading to the cleavage of poly ADP-ribose-polymerase (PARP) and apoptotic events (Boulares et al., 1999).

On the other hand, the phosphatidylinositol 3-kinase (PI3K)/Akt signaling pathway essentially involved in cell survival and the intensified guard of cancer cells from apoptosis during tumorigenesis (Yang et al., 2005; Castaneda et al., 2010). In addition to the PI3K/Akt pathway, mitogen-activated protein kinases (MAPKs) belong to serine–threonine protein kinases, which consist of growth factor-regulated extracellular signal-related kinases (ERKs), c-jun NH2-terminal kinases (JNKs), and p38 MAPKs, and take a great part in regulating various cellular processes, such as cell growth and proliferation, differentiation as well as apoptosis (Boutros et al., 2008). Many researches have indicated that agents interfering the reactive oxygen species (ROS) metabolism can selectively eradicate the cancer cells by elevating the accumulation of ROS above a threshold of toxicity. As cancer cells usually have higher concentration of endogenous ROS as compared with the normal counterpart, the toxic threshold can be easily attained in cancer cells as compared to normal ones (Schumacker, 2006; Fruehauf and Meyskens, 2007). Preceding studies have also suggested that ROS can elicit the activation of the PI3K/Akt and MAPK signal transduction pathways (Torres and Forman, 2003; McCubrey et al., 2006), and the activation of ERK, JNK, and p38 MAPK signaling pathways perform a vital role in growth arrest and apoptosis of cells via generation of ROS (Assefa et al., 2000; El-Najjar et al., 2010).

Naturally occurring compounds, which have been an important arsenal of promising cancer chemotherapeutic and chemopreventive pharmaceuticals, have attracted vast attention due to their assumptive potential to have a wider safety margin (Lee, 2010). Brucein D (BD) is a major active quassinoid isolated from Bruceae Fructus, the fruits of *Brucea javanica* (L.) Merr (Simaroubacae). In our previous work, BD was found to induce apoptosis in PanCa cell line PANC-1 through the activation of ROS-mediated p38-MAPK and inhibition of NF-κB pathway *in vitro* (Lau et al., 2009). The *in vivo* anti-PanCa effect of BD was investigated in a subcutaneous xenograft mouse model (Lau et al., 2010). However, the in-depth *in vitro* and *in vivo* anti-PanCa mechanism remained to be explored.

In the present study, more PanCa cell lines and GES-1 cells were employed for the more detailed *in vitro* study. For the *in vivo* investigation, an EGFP-luciferase-transfected orthotopic tumor mouse model of PanCa was employed. Compared with the subcutaneous xenograft mouse model, the orthotopic model of PanCa can characterize the cellular and molecular pathology of PanCa with increased clinical relevance, recapitulating the human pancreatic tumorigenesis more closely.

Our result gained further insight into the time- and dose-dependent anti-PanCa effect of BD, and provided pioneering evidence that BD significantly suppressed the tumor growth, inhibited the proliferative index and induced caspases/mitochondria-dependent apoptosis through suppressing the activation of PI3K/Akt and MAPKs both *in vivo* and *in vitro*. For the first time, BD was found to exhibit similar anti-PanCa activity as the current first-line agent gemcitabine/5-FU with much smaller dosage and favorable safety profile. This was the first investigation reporting the PI3K/Akt inhibitory effect of quassinoids both *in vivo* and *in vitro*, and also the innovative study exploring the anti-cancer activity and mechanism of C_20_ quassinoids in the orthotopic model of PanCa.

The results provided novel insight into the anti-PanCa effect of this naturally occurring tetracyclic triterpene quassinoid, which further corroborated the modern application of *Brucea javanica* and contributed to its anti-PanCa pharmacological validation. The promising anti-PanCa activity of BD suggests that it holds a promising potential to be developed into a novel effective and safe therapeutic agent for the PanCa chemotherapy.

## Materials and Methods

### Cell Lines and Reagents

Human PanCa cell lines PANC-1, Capan-1, Capan-2, and SW-1990 and non-tumorigenic human gastric epithelial cells GES-1 were purchased from the American Type Culture Collection (ATCC, Manassas, VA, United States). All reagents for cell culture were obtained from Invitrogen, United States. The antibodies against Akt, p-Akt (ser473), p-Akt (thr308), ERK1/2, p-ERK1/2, p38, p-p38, JNK, p-JNK, p-PI3K (Tyr458), PI3K and HRP-conjugated secondary antibodies were purchased from Cell Signaling Technology (Beverly, MA, United States). All other antibodies employed in the present work were provided by Santa Cruz Biotechnology (Santa Cruz, CA, United States) unless otherwise stated. BD was isolated from *B. javanica* fruits (Bruceae Fructus, Ya-Dan-Zi in Chinese) in our laboratory and its structure was elucidated by comparison with the published ESI-MS/MS, UV, ^1^H and ^13^C-NMR spectral data (Lee et al., 1979; Zhao et al., 2011). GEM was purchased from Ely Lilly (Fegersheim, France) and 5-fluorouracil (5-FU) was obtained from Sigma (St. Louis, MO, United States).

### Cell Culture

Capan-1 cells were cultured in Iscove’s Modified Dulbecco’s Medium (IMEM; Gibco, Rockville, MD, United States) which was supplemented with FBS (20%), penicillin (100 U/mL), and streptomycin (100 mg/mL). PANC-1, Capan-2, SW-1990, and GES-1 cells were cultured in Dulbecco’s modified Eagle’s medium (DMEM; Gibco, Rockville, MD, United States) supplied with FBS (10%), penicillin (100 U/mL), and streptomycin (100 mg/mL). Cells were incubated in a 5% CO_2_, 95% humidified atmosphere at 37°C.

### Cell Viability and Apoptosis Assay

The viability of cells was determined by the 3-(4,5-dimethylthiazol-2-yl)-2,5-diphenyltetrazolium bromide assay (MTT; Invitrogen Life Sciences, Carlsbad, CA, United States) as previously described (Lau et al., 2009). Apoptosis was evaluated by using (i) Hoechst 33342 (Invitrogen, Carlsbad, CA, United States) staining or (ii) Cell Death Detection ELISA kit (Roche, Palo Alto, CA, United States) or (iii) Dead Cell Apoptosis Kit with Annexin V Alexa Fluor^®^ 488 and Propidium Iodide (PI) (Invitrogen, Carlsbad, CA, United States) following the protocol outlined by the manufacturers.

### Cell Cycle, ROS, and Mitochondrial Membrane Potential Analysis by Flow Cytometry

Reactive oxygen species levels, mitochondrial transmembrane potential (ΔΨm) and cell cycle analysis were determined with the probe 5-(and-6)-chloromethyl-20, 70-dichlorodihydrofluorescein diacetate (CM-H2DCFDA, Invitrogen, Carlsbad, CA, United States), rhodamine 123 (Rh123, Sigma, St. Louis, MO, United States) and FxCycle^TM^ PI/RNase Staining Solution (Molecular Probes, Eugene, OR, United States) according to the manual outlined by the manufacturer and as previously described (Park et al., 2011), respectively.

### Cytosolic Extracts Preparation

Briefly, after BD treatment, cells were obtained, washed, and resuspended in ice-cold membrane lysis buffer [10 mM HEPES, pH 7.8, 1.5 mM MgCl_2_, 10 mM KCl, 0.2 mM EDTA, 1 mM DTT, 1 mM phenylmethylsulfonyl fluoride (PMSF), 1 mM Na_3_NO_4_, 1% Protease Inhibitor Cocktail]. After incubation on ice for 20 min, the mixture was subjected to centrifugation for 10 min at 15,000 × *g*. The supernatant (the cytosolic extract) was collected and subjected to Western blotting analysis.

### Lentiviral Vector Transduction, Green Fluorescent (EGFP), and Luciferase Activity Determination

The lentivirus (Plent-CWV-EGFP-linker-Luc-PGK-Puro) from the Neuron Biotech Co. (Shanghai, China) was used to transfect Capan-2 cell lines (Detail is provided in Supplementary Methods). After transfection, the cells expressing EGFP were directly observed by confocal microscope (Carl Zeiss GmbH, Jena, Germany). Transfection efficiency was also evaluated by flow cytometer (Beckman Coulter, Fullerton, CA, United States). Additionally, Luc activities in transfected Capan-2 cell lines were detected using a dual-luciferase reporter assay system based on the manufacturer’s manual (Promega) by Luminometer (Infinite M1000, TECAN, Switzerland).

### Orthotopic Implantation of Capan-2 Cells

Athymic nu/nu mice (male, 5–7 weeks old) were supplied by the Laboratory Animal Services Centre of The Chinese University of Hong Kong. The protocol for the whole experiment were reviewed and approved by the Hong Kong Special Administrative Region Department of Health [(13–55) in DH/HA&P/8/2/1 Pt.28] and the Animal Experimentation Ethics Committee of The Chinese University of Hong Kong (Ref. 11/050/GRF-5).

The orthotopic mouse models was established by pancreatic inoculation of Capan-2 cells transfected with *EGFP-Luc* marker genes using the methods described above. EGFP-Luc-transfected Capan-2 cells were harvested and resuspended in PBS. Suspensions comprising single cells with viability above 90% were employed for the following injection operation. Post anesthetization with ketamine–xylazine (10–100 mg/kg) solution, a 1-cm wide incision was made in the left upper quadrant of the abdomen, and Capan-2 (2 × 10^6^) in PBS (100 μL) were injected into the subcapsular region of the pancreas tail with a 29-gauge needle (Terumo BCT Co., Japan). The abdominal muscle layer was subjected to closure with continuous 4-0 suture, and the overlying skin was closed with a second set of interrupted 6-0 vicryl sutures (Jinhuang Medicine Products Co., Shanghai, China). The negative control group was constituted by eight mice which received only Matrigel (60 μL). After recovering from the effects of the anesthetics, all mice were placed under a heat lamp and the temperature, skin color and respiratory rate were monitored. Once in the sterna position, they responded to the external stimuli and freely moved about inside the cage.

### Experimental Protocol

One week post implantation, mice were divided into five groups at random (*n* = 8 each group) according to the bioluminescence firstly detected with an In-Vivo MS FX Pro Imaging System (Bruker BioSpin, Woodbridge, CT, United States) as follows: (a) untreated control (vehicle only; DMSO:saline mixture, 3:10,000, once daily); (b) GEM alone (100 mg/kg, twice weekly); (c) 5-FU alone (50 mg/kg, twice weekly); (d) BD1 alone (1.5 mg/kg, once daily); (e) BD2 alone (0.75 mg/kg, once daily). Administration was given by intraperitoneal injection, and the dosages of BD were employed based on the previous investigation and our preliminary experiment (Lau et al., 2010) (**Figure 6A**).

Tumor volumes of the mice were monitored weekly with the In-Vivo MS FX Pro Imaging system on 0, 7, 14, 21, and 28 days of treatment. Body weights were recorded twice weekly and animals were monitored daily. Treatment was continued for 4 weeks, and euthanization was performed 1 week later. After mouse was euthanized, blood was collected and was immediately subjected to centrifugation at 10,000 rpm for 12 min to produce plasma, and frozen at -80°C for further analysis. Heart, liver, kidneys, and tumors were excised, washed with saline and frozen over dry ice prior to storage at -80°C. The tumor volume was determined as 4/3π (a/2^∗^b/2^∗^c/2), where a, b, c is the mean of the length, width, and depth, respectively. Half of the tumor tissue was fixed in formalin and embedded in paraffin for immunohistochemical analysis and routine hematoxylin and eosin (H&E) staining. The other half was snap frozen in liquid nitrogen.

### Immunohistochemical and Immunofluore Analysis of Ki-67 and PCNA in Tumor Samples

Immunohistochemical analysis was performed on a thick (5 μm), paraformaldehyde-fixed, paraffin-embedded tumor tissue sections using the Mouse and Rabbit Specific HRP/DAB (ABC) Detection IHC Kit (Abcam Inc., Cambridge, United Kingdom) as reported precedingly (Wang et al., 2012). Briefly, tissue sections were immunostained with an anti-Ki-67 (1:50) or anti-PCNA (dilution 1:200). The number of Ki-67 or PCNA positive cells was determined in 10 randomly selected microscopic fields at ×400 magnification. The proliferation index of Ki-67 or PCNA was calculated as: the number of Ki-67 or PCNA positive cells/the total cell count × 100%. Immunofluore analyses for Ki67 (dilution 1:50) and PCNA (dilution 1:200) were carried out with the Vecta Elite kit (Vector Laboratories, Burlingame, CA, United States) based on the manufacturer’s manual. Sample analysis and image acquisition were achieved by Axio Scope A1 fluorescence microscope which was equipped with AxioCam MRC digital camera (Carl Zeiss, Oberkochen, Germany).

### TUNEL Assay for the BD-Induced Apoptosis

The apoptotic tumor cells from tumor tissues were detected by Terminal deoxynucleotidyl transferase dUTP nick end labeling (TUNEL) method using the In Situ Cell Death Detection kit, POD, following the manufacturers’ protocol and as previously described (Wang et al., 2012). The DNA strand breaks during apoptosis were detected under a light microscope (Olympus IX71, Japan). The proportion of TUNEL-positive cells was determined in 10 randomly selected microscopic fields at ×400 magnification.

### Western Blot Analysis

Cells and tumor tissues were lysed with RIPA buffer consisting of protease inhibitor cocktail (Roche Molecular Biochemicals, Switzerland), 1 mM PMSF and 1 mM Na_3_NO_4_. Then protein level was measured by a bicinchoninic acid (BCA) assay (BCA kit, Sigma–Aldrich, St. Louis, MO, United States). Proteins were then subjected to fractionation by SDS-polyacrylamide gel electrophoresis, electrotransferred to polyvinylidene fluoride (PVDF) membrane (Immobilon, Millipore, United States), blotted with corresponding antibodies, and then detected by enhanced chemiluminescence (NOVEX, San Diego, CA, United States). The immunoreactive band intensity was quantified by ImageJ software (National Institutes of Health, United States).

### Measurement of Plasma-Specific Enzyme Levels

The concentrations of aspartate aminotransferase (AST) and alanine aminotransferase (ALT) as index for hepatic function, lactate dehydrogenase (LDH) and creatine kinase (CK) for the assessment of heart status, and creatinine indicative of kidney function were determined using commercially available kits (Abcam Inc., Cambridge, United Kingdom) according to the manufacturer’s protocol.

### Statistical Analysis

Data are expressed as means ± SD from three independent experiments. The values were analyzed by one-way analysis of variance (ANOVA) and an unpaired Student’s *t*-test. Figures were produced using SigmaPlot 12 software package (Systat Software, Inc.). Differences were deemed to be statistically significant when *P* < 0.05.

## Results

### The Anti-proliferative Effects of BD in Human PanCa Cells

**FIGURE 1:**
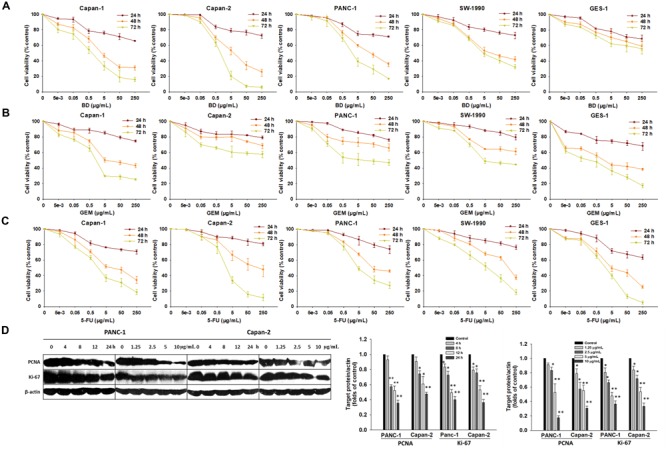
Anti-proliferative effects of BD, GEM, and 5-FU on PanCa cells and non-tumorigenic GES-1 cell lines. **(A–C)** Cells were treated with either increasing concentrations of BD **(A)**, GEM **(B)**, or 5-FU **(C)** for 24, 48, and 72 h and subjected to MTT assay. **(D)** The protein expression of PCNA and Ki-67 was detected by Western blotting after different doses of BD treatments for 12 h, and 5 μg/mL of BD treatment for 4–24 h. β-Actin served as the protein loading control. Each bar represents means ± SD of three separate experiments. ^∗^*P* < 0.05 and ^∗∗^*P* < 0.01 vs the control group.

Several PanCa cell lines and GES-1 (non-malignant) were employed to evaluate the potential cytotoxicity of BD and the clinical first-line drugs, GEM and 5-FU. As shown in **Figures 1A–C** and Supplementary Table S1, cell growth was markedly inhibited by BD treatment in a dose- and time-related fashion. BD was shown to exhibit more potent cytotoxic effect on PANC-1, Capan-2, and Capan-1 cells except for SW-1990 cells, when compared with GEM and 5-FU though the latter two chemicals also showed satisfactory *in vitro* anti-PanCa effects. It is noteworthy that BD only exerted mild cytotoxic effect on human normal GES-1 cells with IC50 value > 200 μg/mL, while GEM and 5-FU had stronger cytotoxic effects on GES-1 cells with IC50 values of 0.13 μg/mL and 1.66 μg/mL, respectively. Moreover, we evaluated the protein expressions of PCNA and Ki-67, two important markers of cell proliferative activity, in PANC-1 and Capan-2 cells subjected to BD treatment. As shown in **Figure 1D**, BD treatment led to significant down-regulation of PCNA and Ki-67 expressions in a time- and dose-related fashion in both PANC-1 and Capan-2 cell lines.

### BD Induces Apoptosis and S Phase Arrest in PANC-1 and Capan-2 Cells

**FIGURE 2:**
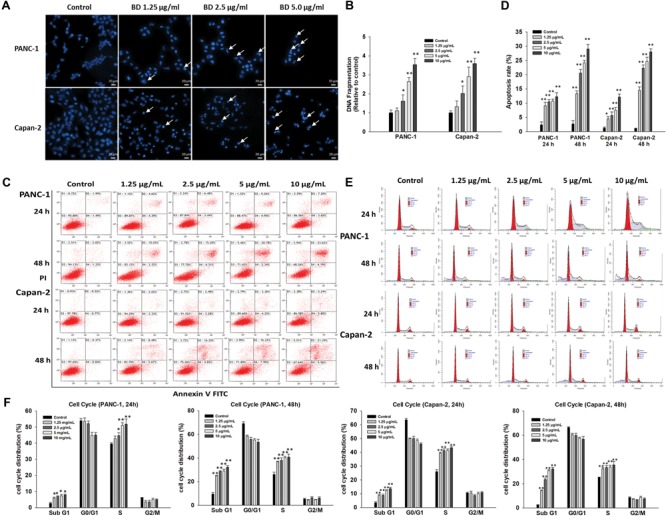
BD elicits apoptosis and S-phase arrest in PANC-1 and Capan-2 cells. **(A)** PANC-1 and Capan-2 cells were subjected to cell nucleus morphological analysis using Hoechst 33342 after different concentrations of BD (1.25, 2.5, and 5 μg/mL) treatments for 48 h. Representative fluorescence images of Hoechst 33342 positive cells are shown. Scale bar, 20 μm. **(B)** Cells were treated with various concentrations of BD (1.25, 2.5, 5, and 10 μg/mL) for 72 h, followed by Cell Death Detection ELISA assay. **(C,D)** Cells were exposed to different concentrations of BD (1.25, 2.5, 5, and 10 μg/mL) for 24 and 48 h, followed by flow cytometry apoptosis assay. Bar graphs show the proportion of early and late apoptotic cells. **(E,F)** Cells were treated with BD (1.25, 2.5, 5, and 10 μg/mL) for 24 and 48 h, and the cell cycle phases were evaluated by flow cytometry. Each bar represents means ± SD of three separate experiments. ^∗^*P* < 0.05 and ^∗∗^*P* < 0.01 vs the control group.

To decipher whether BD-mediated cytotoxic effect on PANC-1 and Capan-2 cells were associated with the induction of apoptosis or necrosis, Hoechst 33342 nuclear staining, Cell Death Detection ELISA, Annexin V-PI staining and flow cytometric assay were carried out. As shown in **Figure 2A** and Supplementary Figure S1, all BD treatment groups exhibited typical apoptotic features such as cellular shrinkage, cell wall blebs, apoptotic bodies, and phosphatidylserine exposure in a dose-related manner. As depicted in **Figure 2B**, the DNA fragmentation increased with the ascending concentrations of BD, and statistically significant differences were achieved between control and BD-treated groups (BD concentration ≥ 2.5 μg/mL). To further ascertain the apoptotic effects, flow cytometry was employed. The Annexin V-FITC/PI staining results also indicated that BD remarkably elicited apoptosis in PANC-1 and Capan-2 cells in a dose- and time-related fashion (**Figures 2C,D**). Cell cycle distributions of PANC-1 and Capan-2 cells were also detected by flow cytometry. BD treatment resulted in a marked accumulation of S-phase cells in a dose- and time-related manner. Moreover, treatment with BD of a higher concentration or a longer exposure significantly elevated the percentage of cells at the sub-G1 phase, suggestive of apoptotic peaks in a dose- and time-related manner (**Figures 2E,F**). Taken together, these results were consistent with each other, and further corroborated that BD treatment led to the cell cycle arrest at the S phase and elicited apoptosis in both PANC-1 and Capan-2 cells in a dose- and time-related fashion.

### BD Activates the Caspase-Dependent Pathway and Induces Cellular Apoptosis

**FIGURE 3:**
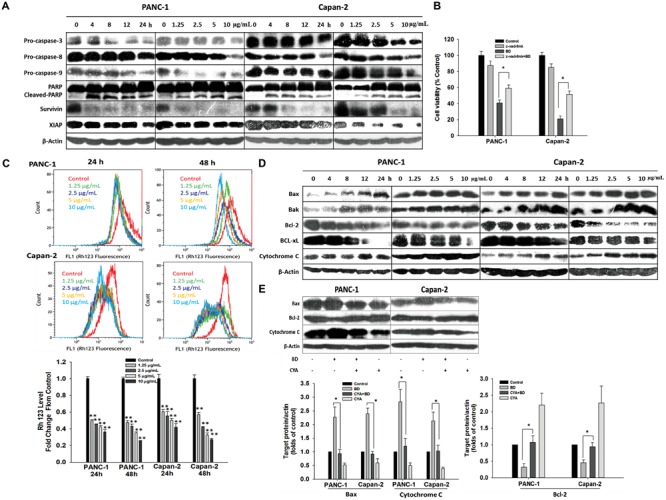
BD triggers caspases/mitochondria-dependent apoptosis. **(A)** Cells were treated with BD either at concentrations of 1.25, 2.5, 5, and 10 μg/mL for 12 h or at 5 μg/mL for various durations of 4, 8, 12, and 24 h. The cell lysates harvested were analyzed for expression of PARP-1, pro-caspase-3, pro-caspase-8, pro-caspase-9, Survivin and XIAP by Western blotting. β-Actin served as the protein loading control. **(B)** Z-VAD-FMK (caspase inhibitor, 50 μM) was added simultaneously with BD to PANC-1 and Capan-2 cells. Cells were harvested for cell viability analysis by MTT assay. **(C)** Cells were treated with BD at the indicated concentrations for 24 and 48 h and were stained by Rhodamine 123 to determine the change of ΔΨm by flow cytometry. **(D)** Protein expression involved in the mitochondrial function were measured by Western blotting in both PANC-1 and Capan-2 cells treated with various concentrations of BD for 12 h or at 5 μg/mL for different treatment durations. β-Actin served as the protein loading control. **(E)** Western blotting analysis of Bax, Bcl-2, and cytochrome c levels after cells were incubated with cyclosporine A (10 μM) or BD (5 μg/mL) alone or in combination for 24 h. β-Actin served as the protein loading control. Each bar represents means ± SD of three independent experiments. ^∗^*P* < 0.05 and ^∗∗^*P* < 0.01 vs the control group.

To dissect the possible underlying mechanism by which BD elicited apoptosis in PANC-1 and Capan-2 cells, the effect of BD on the activation of different caspases, vital contributors of apoptosis, was determined. As depicted in **Figure 3A** and Supplementary Figure S2, the Western blotting assay showed an up-regulated expression level of cleaved-PARP (89 kDa proteolytic fragments) in BD-treated cells. Downregulated expression levels of pro-caspase-9, pro-caspase-3, pro-caspase-8, Survivin and XIAP after BD treatment were also observed simultaneously. These observations suggested that BD could activate the caspase-dependent pathway. To further verify these results, the potential role of caspases in BD-elicited cellular apoptosis was explored. The data suggested that pretreatment with the pan-caspase inhibitor z-VAD-fmk substantially attenuated the BD-elicited cell death in PANC-1 and Capan-2 cells (**Figure 3B**), indicating that the BD-elicited apoptosis in PanCa cells was closely associated with the caspase-dependent pathway.

### BD Triggers Mitochondria-Dependent Apoptosis

The cell mitochondrial membrane potential (MMP) collapse causes mitochondrial dysfunction leading to swelling, release of cytochrome c and apoptosis. Therefore, whether BD treatment induced alternations in the MMP was examined using rhodamine 123 (Rh123) dye. As shown in **Figure 3C**, the accumulation of Rh123 in the mitochondria was notably decreased in BD treatment in comparison with the non-treated cells in a time- and dose-dependent fashion. It has been established that the Bcl-2 family members regulate the mitochondrial cell death pathway; particularly, pro-apoptotic Bax and anti-apoptotic Bcl-2 are important for the cytochrome C release and the subsequent downstream activation of caspase protein (Su et al., 2014). In the present work, the expression of several mitochondrial-associated apoptotic proteins (Bcl-2, Bcl-xL, Bak, Bax, and cytochrome C), caspase proteins (caspase-3 and caspase-9) and PARP was determined by Western blotting. The expression of Bax, Bak, and cytosolic cytochrome C was substantially elevated in a dose- and time-related fashion in the two BD-treated PanCa cells. By contrast, the expression of Bcl-2 and Bcl-xL was obviously decreased upon BD treatment (**Figure 3D** and Supplementary Figure S2). Meanwhile, caspase-9, caspase-3, and PARP were shown to be observably activated (**Figure 3A** and Supplementary Figure S2).

These results amply suggested that the BD-elicited cellular apoptosis was related to the activation of the mitochondrial-associated pathway in PANC-1 and Capan-2 cells. To further confirm these results, the mitochondrial permeability transition inhibitor (cyclosporine A; CyA) was used prior to treatment with BD in PANC-1 and Capan-2 cells, and the expression of several mitochondrial-associated apoptotic proteins, such as cytosolic cytochrome C, Bax and Bcl-2, was analyzed by Western blotting. The expression level of Bcl-2 was upregulated in BD-treated cells when pretreated with CyA. By contrast, the Bax and cytosolic cytochrome C expression was markedly downregulated in BD-treated cells when pretreated with CyA (**Figure 3E**). To sum up, these results indicated that the BD-induced PanCa cellular apoptosis might involve the mitochondria dysfunction-mediated apoptotic pathway.

### The PI3K/Akt Signal Pathway Is Involved in BD-Induced PanCa Apoptosis

**FIGURE 4:**
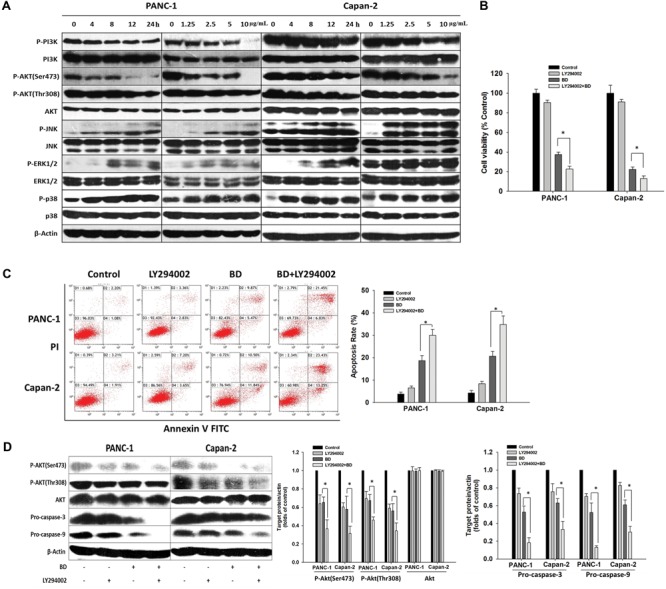
The PI3K/Akt signal pathway is involved in BD-elicited PanCa apoptosis. **(A)** Cells were treated with various concentrations of BD (1.25, 2.5, 5, and 10 μg/mL) for 12 h or BD at 5 μg/mL for 4, 8, 12, and 24 h. Cells were harvested and then analyzed for the expression of PI3K/Akt and MAPK pathways-related proteins by Western blotting. β-Actin served as the protein loading control. **(B)** Cells were treated with BD for 72 h alone or in combination with pretreatment of 50 μM LY-294002 for 1 h, respectively, and subjected to cell viability assay by MTT. **(C)** Cells were pretreated with LY-294002 (50 μM) for 1 h, followed by 5 μg/mL BD for 48 h. Flow cytometric analysis was performed by Annexin V-FITC and PI double-staining. **(D)** Cells were incubated with LY-294002 (50 μM) for 1 h prior to BD treatment for another 12 h. The same amount of cell lysates was analyzed by Western blotting using antibodies against p-Akt, Akt, pro-caspase-3, and pro-caspase-9. β-Actin served as the loading control. Each bar represents means ± SD of three independent experiments, ^∗^*P* < 0.05.

The protein expression involved in the PI3K/Akt and MAPKs signal pathways was determined to illuminate their potential roles in BD-induced apoptosis. As exhibited in **Figure 4A** and Supplementary Figure S3, the expression of phosphorylated p38, ERK1/2, and JNK were remarkably upregulated in a dose- and time-related fashion. By contrast, the expression of non-phosphorylated PI3K, Akt, ERK, JNK, and p38 was not significantly altered upon BD treatment, nor for treatment with 5 μg/mL BD for different durations. Moreover, the decreased phosphorylation of PI3K, Akt (Ser473), and Akt (Thr308) was also shown in BD-treated cells dose- and time-dependently.

Next, to explore whether BD-elicited human PanCa apoptosis was mediated by the decrease of PI3K/Akt kinase activity, PANC-1, and Capan-2 cells were treated with BD in combination with LY294002 (PI3K inhibitor) and the cell viabilities were determined with MTT assay post combined treatment for 72 h. The result indicated that BD and LY294002 synergistically elicited apoptosis in both PANC-1 and Capan-2 cells (**Figure 4B**), suggesting that PI3K/Akt was potentially involved in BD-induced human PanCa apoptosis. To confirm this mechanism, flow cytometric analysis and Western blotting were employed simultaneously. As expected, the data suggested that apoptosis was accentuated by LY294002 pretreatment when compared with that induced by BD monotherapy (**Figure 4C**). Moreover, when the PI3K/Akt signaling was significantly suppressed by LY294002, the expression of pro-caspase 3 and pro-caspase 9 was synergistically attenuated in both PANC-1 and Capan-2 cells treated with BD (**Figure 4D**). The result was congruent with that of the MTT assay. Therefore, these results indicated that the BD-induced PANC-1 and Capan-2 cellular apoptosis were mediated, at least partially, by inhibition of the PI3K/Akt signal pathway.

### Accumulation of ROS Is a Critical Event in BD-Induced Cellular Apoptosis

**FIGURE 5:**
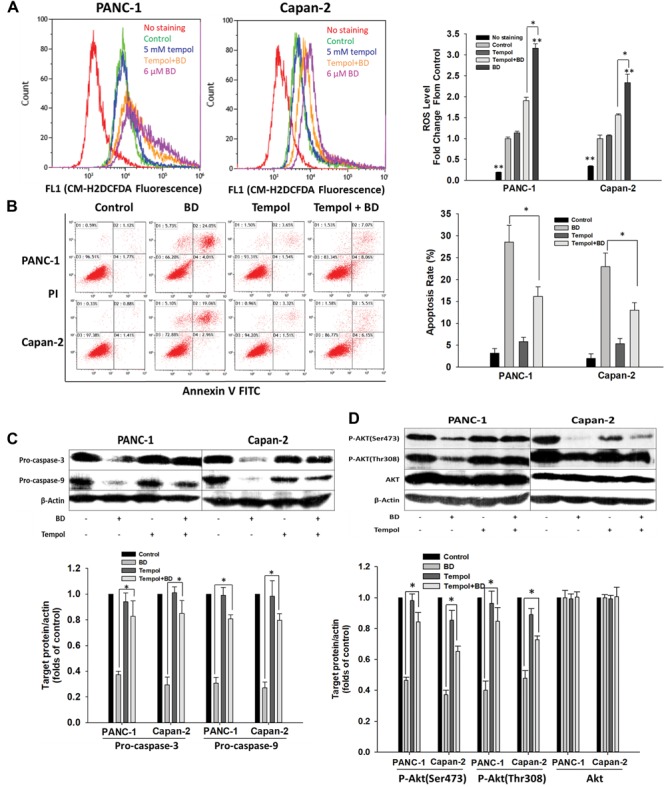
Brucein D-mediated apoptosis involves ROS generation. **(A)** Representative flow cytometry histograms showing the relative content of ROS after pretreatment with tempol (5 mM) for 2 h, followed by 12 μM BD treatment for 24 h. Data are expressed as fold changes in CM-H2DCFDA fluorescent levels of the treated cells to the non-treated cells (Control). **(B)** Flow cytometric analysis was carried out by Annexin V-FITC and PI double-staining after pretreatment with tempol (5 mM) for 2 h, followed by 12 μM BD treatment for 48 h. **(C)** Protein lysates of the cells either pretreated with 5 mM tempol for 2 h or left alone followed by BD treatment for 24 h were analyzed by Western blotting. β-Actin served as the loading control. **(D)** Cells were either pretreated with 5 mM tempol for 2 h or left alone followed by BD treatment for 24 h. The expression of Akt and p-Akt was analyzed by Western blotting. β-Actin served as the loading control. Each bar represents means ± SD of three separate experiments. ^∗^*P* < 0.05 and ^∗∗^*P* < 0.01 vs the control group.

To elucidate whether BD triggered ROS accumulation in PanCa cells, the change of intracellular ROS was detected using the cell-permeable dye (CM-H2DCFDA). As shown in **Figure 5A**, the intensity of FITC channel was increased (peak was right-shifted) in BD-treated cells, implying that treatment with BD caused elevation of ROS level in PANC-1 and Capan-2 cells compared with untreated cells. The intracellular ROS levels were observed to be appreciably elevated in BD-treated PANC-1 and Capan-2 cells, indicating that production of ROS was possibly associated with the apoptosis of PanCa cells elicited by BD.

To investigate whether ROS generation was related to the BD-elicited cellular apoptosis, cells were treated with BD in the presence or absence of tempol. It was observed that pretreatment with tempol inhibited the accumulation of ROS provoked by BD (**Figure 5A**). Intriguingly, flow cytometric assay suggested that apoptosis was significantly ameliorated by tempol pretreatment in comparison with the that elicited by BD treatment alone (**Figure 5B**). These findings suggested the accumulation of ROS might be involved in the BD-elicited cellular apoptosis. Moreover, the role of ROS in the expression of apoptosis-related proteins was analyzed. Western blotting assay indicated that BD decreased the expression of pro-caspase-3 and pro-caspase-9 in PanCa cells, and the effects were compromised by pretreatment with tempol prior to BD treatment (**Figure 5C**). These observations further indicated that ROS took a vital part in BD-elicited cellular apoptosis in PANC-1 and Capan-2 cells. Furthermore, the possible effect of ROS-mediated PI3K/Akt pathway in BD-treated PanCa cells was investigated. PANC-1 and Capan-2 cells were treated with 5 μg/mL BD for 24 h alone or in combination with pretreatment with 5 mM tempol for 2 h and then analyzed by Western blotting for Akt expression. The results indicated that, after BD treatment, intracellular ROS was accumulated (**Figure 5A**) and p-Akt protein expression was decreased (**Figure 5D**). However, with the decrement of ROS by tempol, intracellular p-Akt protein expression was observably recovered even with BD treatment (**Figure 5D**). The results suggested that ROS generation was required for the PI3K/Akt signaling pathway in BD-induced cellular apoptosis in human PanCa cells.

### BD Suppresses the Tumor Progression in Orthotopic Xenograft Mouse Model

**FIGURE 6:**
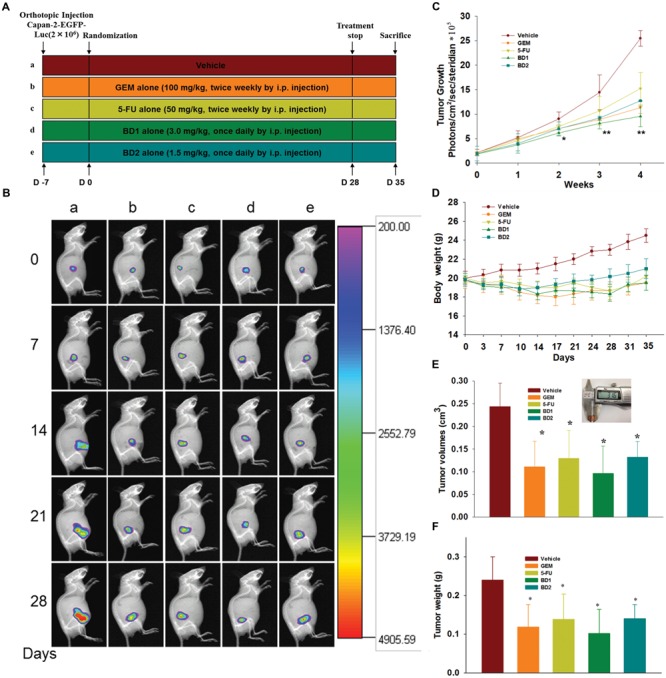
BD inhibits the tumor progression in orthotopic xenograft mouse model. **(A)** Schematic depiction of the experimental schedule as presented in section “Materials and Methods” (*n* = 8). **(B)** Bioluminescence In-Vivo FX PRO images of orthotopically implanted Capan-2 cells in live, anesthetized mice under different treatment regimens every week. **(C)** Determination of photons per second showing the tumor volume at different time points by live bioluminescence imaging. **(D)** Body weight alternations of mice were determined at the indicated time. **(E)** Tumor volumes in mice were determined at autopsy with vernier calipers and calculated following the formula V = 4/3π(a/2^∗^b/2^∗^c/2) (*n* = 8). **(F)** Average tumor weight of each group on the last day of the experiment. Each column represents the mean ± SD of eight samples. ^∗^*P* < 0.05 and ^∗∗^*P* < 0.01 vs. the control group.

Based on our promising *in vitro* results, we further analyzed the *in vivo* therapeutic effect of BD on the growth of orthotopically implanted human PanCa cells in nude mice. The experimental protocol is exhibited in **Figure 6A**. Capan-2 cells were used for the *in vivo* studies since Capan-2 was relatively more sensitive and was stably transfected with EGFP and luciferase (Supplementary Figure S4). Dynamic growth and remote metastasis of PanCa in mice were monitored weekly by bioluminescence imaging. As shown in **Figures 6B,C**, the tumor volumes in control mice were significantly larger than those of mice from the rest groups since day 14. On the 35th day, mice were euthanized and tumors were obtained to measure the volume and weight. The results were congruent with those from the bioluminescence imaging, indicating that BD treatment significantly suppressed the growth of tumor in comparison to the control (**Figures 6E,F**). Furthermore, BD at low dose (1.5 mg/kg) was shown to exert similar anti-tumor effect as GEM, suggestive of the appreciable anti-PanCa potency of BD. Serum biochemical parameters were not altered significantly among the control and treatment groups, indicating that there probably was no obvious toxicity induced by BD treatment (Supplementary Table S2). We further studied the distant organ metastasis in mice. After mice were sacrificed, the whole body and vital organs were imaged using the In-Vivo MS FX PRO Imaging System. No EGFP-labeled tumors were found in organs except the pancreas for all the vehicle- and drug-treated mice (Supplementary Figure S5).

**FIGURE 7:**
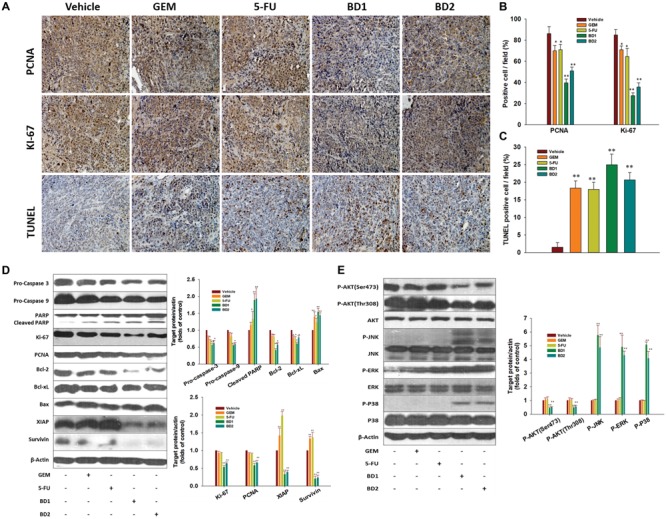
BD inhibits the proliferative index and elicits apoptosis in orthotopic xenograft mouse model. **(A–C)** The representative tumor tissue sections from xenografts in different treatment groups were analyzed by immunohistochemical analysis for the protein expression of the proliferation markers PCNA and Ki-67. The apoptotic cells were stained by TUNEL agent. Scale bar, 20 μm **(A)**. Quantification of PCNA/Ki-67/TUNEL positive cells **(B,C)**. A total of 10 fields (40×) were examined and stained cells were determined in four tumor tissues from each group. Each column represents the mean ± SD of four samples. ^∗^*P* < 0.05 and ^∗∗^*P* < 0.01 vs. the control group. **(D)** Western blotting analysis showed the expression of procaspase-3, procaspase-9, PARP, Ki-67, PCNA, Bcl-2, Bcl-xL, Bax, Survivin, and XIAP detected in different treatment groups. β-Actin served as the protein loading control. **(E)** Western blotting analysis on the expression of PI3K/Akt and MAPK pathways-related proteins from respective tumoral homogenate, with β-actin serving as the protein loading control. Data are shown as the mean ± SD from three independent experiments, ^∗^*P* < 0.05 and ^∗∗^*P* < 0.01 vs. the control group.

To further dissect the potential mechanisms underlying the tumor-suppressive activities of treatments, the expression of cell proliferation markers PCNA and Ki-67 in tumor tissues from the mice of different treatment groups was examined. The results in **Figures 7A,B** showed that BD significantly decreased the expression of PCNA and Ki-67 in tumor tissues relative to the control, and similar trend was found in immunofluorescence analysis (Supplementary Figure S6). Furthermore, the expression of PCNA and Ki-67 in tumor lysates was further supported by Western blotting (**Figure 7D**), indicating BD could dramatically inhibit the proliferation of tumor cells.

### BD Induces Apoptosis and Suppresses Activation of PI3K/Akt in Pancreatic Tumor Tissues

We further investigated whether the pancreatic tumor growth inhibition by BD in our model was related to the induction of apoptosis, inactivation of PI3K/Akt and activation of MAPKs. The *in situ* TUNEL assay was employed to detect the apoptotic cells in the tumor tissues. The results indicated that the number of TUNEL positive cells was remarkably elevated in tumors from treatment groups compared with control (**Figures 7A,C**). Western blotting results showed that BD appreciably downregulated the expression of all the tested anti-apoptosis proteins (Bcl-2, Bcl-xL, Survivin, XIAP), and upregulated the expression of pro-apoptotic Bax compared with the vehicle treatment in the orthotopic transplantation models. Meanwhile, BD induced the cleavage of PARP-1 in mouse tumors (**Figure 7D**). These findings draw a parallel with the accentuated apoptosis as evidenced by increased TUNEL staining within tumors. Furthermore, the protein expression involved in the PI3K/Akt and MAPKs signal pathways was examined in the tumor lysates by Western blotting. As shown in **Figure 7E**, the protein expression of phosphorylated forms of Akt (Ser473) and Akt (Thr308) proteins was drastically suppressed by BD treatment. Moreover, up-regulated expression of phosphorylated p38, ERK1/2 and JNK was also observed by BD treatment. By contrast, the expression of non-phosphorylated Akt (Ser473), Akt (Thr308), JNK, ERK, and p38 was not significantly altered upon BD treatment. These data suggested that modulation of PI3K/Akt activity might be an important molecular mechanism underlying the *in vivo* anti-PanCa effects exerted by BD.

## Discussion

Quassinoids, degraded triterpenes commonly found in species of Simaroubaceae, are renowned for the anti-cancer activity (Guo et al., 2005). BD is one of the major active quassinoids isolated from Bruceae Fructus which is commonly used for the treatment of cancers in South East Asia (Lee et al., 1984). BD has been shown to exhibit remarkable suppressive effect on the proliferation of PanCa cells (Lau et al., 2009). Our results also indicated that BD, GEM, and 5-FU had mixed potency in eliciting cytotoxicity on the PANC-1, Capan-2, SW-1990, and Capan-1 cell lines. BD was shown to harbor more potent cytotoxic activity as compared to GEM and 5-FU in inhibiting Capan-1, PANC-1, and Capan-2 cell growth, while exhibited less anti-proliferative effect on SW-1990 cells with respective to 5-FU. Nevertheless, BD exerted much less cytotoxicity in the non-tumorigenic GES-1 cells than GEM and 5-FU (**Figures 1A–C** and Supplementary Table S1). Therefore, this compound is believed to have the potential to be further developed into an effective and less toxic therapeutic candidate for PanCa treatment. In this study, it was proved that the BD-elicited cellular apoptosis in human PanCa was intimately related to the activation of ROS and inhibition of PI3K/Akt activation both *in vitro* and *in vivo*.

Apoptosis, a programmed cell death process vital for the survival of organisms, involves series of biochemical events resulting in several pivotal featured morphological alternations, such as membrane blebbing, chromatin condensation, and nucleus DNA fragmentation (Jin and El-Deiry, 2005; Fulda and Debatin, 2006). Our results obtained indicated that BD elicited an obvious increment in nuclei with apoptotic bodies and condensed nuclear, nuclear DNA fragmentation, phosphatidyl-serine externalization, and increased percentage of cells in the sub-G1 phase (**Figures 2A–F** and Supplementary Figure S1).

The extrinsic (cell death receptor-mediated) and the intrinsic (mitochondria-mediated) pathways are two basic apoptotic pathways (Putcha et al., 2002). Activation of caspase-8 triggers the extrinsic pathway (Nagata, 1997) whereas the intrinsic pathway involves permeabilization of mitochondrial outer-membrane and release of pro-apoptotic factors such as cytochrome c from the mitochondria into cytoplasm, subsequently facilitating caspase activation via the cytochrome c/Apaf-1/caspase-9 cascade (Wajant, 2002; Saelens et al., 2004). Both extrinsic and intrinsic pathways eventually culminate in the activation of caspases-3, an essential downstream effector (Li and Yuan, 2008). In the present work, it was found that the BD-mediated S cell cycle arrest played a significant role in the growth inhibition of both PANC-1 and Capan-2 PanCa cells (**Figures 2E,F**). BD significantly downregulated the expression of pro-caspase-8 and pro-caspase-3, and upregulated the expression of cleaved PARP (**Figure 3A**). Meanwhile, the Rh123 accumulation in the mitochondria was significantly decreased upon BD treatment time- and dose-dependently in comparison with the control cells. Additionally, increased expression of pro-apoptotic Bax and Bak and decreased expression of anti-apoptotic Bcl-2 and Bcl-xL were found in BD-treated PANC-1 and Capan-2 cells. The elevated ratio of Bax/Bak: Bcl-2/Bcl-xL would cause the MMP loss and cytochrome c release (**Figures 3C–E**), which might be intimately associated with the activation of caspase-9. These results indicated that BD evoked apoptosis in PANC-1 and Capan-2 cells through both the caspase-8-mediated extrinsic pathway, and the caspase-9-mediated intrinsic pathway, thus subsequently activating the common downstream apoptosis effector caspase-3.

MAPKs, ERKs, JNK, and p38-MAPKs are reported to participate in cell survival, growth, differentiation and apoptosis (Ghobrial et al., 2005). ERK1/2 is generally related to cell survival, whereas JNK and p38 MAPK are elicited by stress responses and cellular apoptosis (Park and Kim, 2005; Wada et al., 2008). In our previous study, the BD-elicited apoptosis in PANC-1 cell line was found to involve the activation of p38-MAPK signaling pathway (Lau et al., 2009). In the current study, it was found that the phosphorylations of p38, ERK, and JNK were significantly upregulated after BD treatment in a dose- and time-related fashion (**Figure 4A** and Supplementary Figure S3). Hence, these results strongly suggested that the BD-elicited cellular apoptosis was closely related to the activation of the MAPKs pathway in PANC-1 and Capan-2 cells.

The PI3K/Akt cell signaling cascade is commonly activated in many types of cancers, regulating cell survival, differentiation, proliferation, and apoptosis (Janku et al., 2012), and the inhibition of Akt phosphorylation has been deemed as a feasible strategy for the therapeutic treatment against human cancers (Hong et al., 2014; Liu et al., 2014). The activation of Akt by PI3K attenuates apoptosis and facilitates tumor cell growth via phosphorylation to inhibit various downstream targets, including the pro-apoptotic Bad, Bax, and caspase-9 (Franke et al., 2003; Liu et al., 2011), as well as transcription factors (Babchia et al., 2010) and other protein kinases (Tang and Lasky, 2003). In the current study, we found that BD dose- and time-dependently inhibited the PI3K/Akt activation. To further verify the role of PI3K/Akt signaling pathway in BD-elicited cellular apoptosis, the typical PI3K inhibitor LY294002 was employed. LY294002 was shown to markedly promote the BD-induced cellular apoptosis, PI3K/Akt inactivation, and up-regulate the pro-caspase-3 and pro-caspase-9 expression, implying that the PANC-1 and Capan-2 cellular apoptosis evoked by BD was mediated, at least partially, via the PI3K/Akt signaling pathway (**Figures 4A–D** and Supplementary Figure S3).

Reactive oxygen species is vital for cell proliferation, differentiation, apoptosis, and survival. Low ROS level is essential in maintaining redox equilibrium and cell proliferation (Sauer et al., 2001). Whereas, excessive accumulation of ROS elicits protein oxidation, lipid peroxidation, cellular DNA damage, and ultimate cell death or apoptosis (Bogurcu et al., 2011; Chen et al., 2013). Previous studies have suggested that ROS produced by several chemotherapeutic agents are vital for eliciting apoptosis in some cancers (Simon et al., 2000; Schumacker, 2006). Consistent with previous report that BD provoked ROS generation in PANC-1 cells (Lau et al., 2010), our present result showed that BD could significantly elevate the intracellular ROS level in PANC-1 and Capan-2 cells. Furthermore, it was shown that pretreatment with tempol led to significant decreases in both intracellular ROS level and BD-elicited cellular apoptosis. Western blotting analysis suggested that pretreatment with tempol inhibited the suppression of pro-caspase-3 and pro-caspase-9 induced by BD in both PANC-1 and Capan-2 cells (**Figures 5A–C**). These findings indicated that accumulation of ROS contributed to the BD-elicited apoptosis in human PanCa cells. It has been established that ROS-mediated cellular apoptosis is regulated by Akt and MAPK signaling pathways in cancers (Ravindran et al., 2011; Yuan et al., 2012). Hence, we proposed that ROS might perform a vital role in regulating the PI3K/Akt signaling pathway in BD-elicited cellular apoptosis, which was supported by tempol application (**Figure 5D**). To test this hypothesis, tempol was employed for the Western blotting assay of p-Akt protein in PANC-1 and Capan-2 cells subjected to BD treatment (**Figure 5D**). The results outlined here unequivocally indicated that ROS generation critically involved in the PI3K/Akt pathway in BD-induced human PanCa cells.

In addition, our *in vivo* studies also indicated that BD effectively suppressed the tumor growth and elicited apoptosis in a EGFP-luciferase-transfected Capan-2 xenograft tumor model without causing mortality or other noticeable side effects. Administration of BD was shown to be as effective as gemcitabine/5-FU in reducing tumor volume, weight and Luc-signal intensity (**Figures 6C–F**). These findings were accompanied by a decreased proliferation and increased apoptosis, as evidenced by PCNA and Ki-67 immunostaining and TUNEL staining with tumor tissues. Moreover, our results indicated that the down-regulation of PI3K/Akt activation, modulation of expression of apoptosis-regulated gene products such as pro-caspase 3, pro-caspase 9, Bcl-2, Bcl-xL, Survivin, XIAP, and the activation of MAPKs were responsible for the therapeutic effects of BD in xenograft model (**Figures 7D,E**). The results also showed that BD administration even at high concentration (3 mg/kg) had no obvious toxicity in mice (Supplementary Table S2). At the same time, no occurrence of distant organ metastasis in mice was found according to the results of bioluminescence (Supplementary Figure S6). These *in vivo* results were in good concert with our *in vitro* investigations, indicating that the modulation of PI3K/Akt activity was possibly one of the molecular mechanisms underlying the inhibitory effect of BD against PanCa.

## Conclusion

Our results suggested that BD could effectively suppress the proliferation and elicit apoptosis in PANC-1 and Capan-2 cells. BD was for the first time found to suppress the tumor growth both *in vivo* and *in vitro* through ROS induction which down-regulated the PI3K/Akt signaling and activation of MAPK pathway, and in turn, activated the caspase-/mitochondrion-mediated pathway including loss of MMP, increment of Bax/Bcl-2 ratio, release of cytochrome c and activation of caspase-3, 8, 9, finally leading to the cleavage of PARP and cellular apoptosis. This was the first investigation reporting the PI3K/Akt inhibitory effect of quassinoids both *in vivo* and *in vitro*. Our pioneering endeavor also indicated that BD exhibited similar anti-PanCa activity as the current first-line agent gemcitabine/5-FU with much smaller dosage and favorable safety profile. These studies are envisaged to provided further insight into the potential of BD as a promising therapeutic agent against PanCa. Positive results from the *in vivo* studies will lay a foundation for future clinical study of this naturally occurring anti-cancer agent.

## Author Contributions

Participated in research design: Z-QL, S-PI, P-SL, and Z-XL. Conducted experiments: Z-QL, S-PI, H-JL, ZL, and J-HX. Contributed new reagents or analytic tools: Z-QL, Z-RS, Y-LC, and Y-FX. Performed data analysis: Z-QL, S-PI, H-JL and ZL. Wrote or contributed to the writing of the manuscript: Z-QL, J-HX, and Z-XL.

## Conflict of Interest Statement

The authors declare that the research was conducted in the absence of any commercial or financial relationships that could be construed as a potential conflict of interest.
