# Supplementary material for: Brucein D, a Naturally Occurring Tetracyclic Triterpene Quassinoid, Induces Apoptosis in Pancreatic Cancer through ROS-Associated PI3K/Akt Signaling Pathway
Source: Front Pharmacol. 2017 Dec 22;8:936. doi: 10.3389/fphar.2017.00936 (PMC5744014; doi:10.3389/fphar.2017.00936)
Supplement: Supplementary file 1 [file Table_1.docx]

**Brucein D, a naturally occurring tetracyclic triterpene quassinoid, induces apoptosis in pancreatic cancer through ROS-associated PI3K/Akt signaling pathway**

***Zheng-Quan Lai ^1^, Siu-Po Ip*** ***^1^, Hui-Jun Liao ^2^, Zheng Lu ^1, 3^, Jian-Hui Xie ^4^, Zi-Ren Su ^5^, Yun-Long Chen ^1^, Yan-Fang Xian ^1^, Po-Sing Leung ^6^, Zhi-Xiu Lin ^1^ ****

*^1^ School of Chinese Medicine, Faculty of Medicine, The Chinese University of Hong Kong, Shatin, N.T., Hong Kong SAR, China*

*^2^ Department of Clinical Pharmacy and Pharmaceutical services, Shenzhen Sixth People's Hospital (Nanshan Hospital), Shenzhen, China*

*^3^ Liver Cirrhosis Diagnosis and Treatment Center, Beijing 302 Hospital, Beijing, China*

*^4^ Guangdong Provincial Key Laboratory of Clinical Research on Traditional Chinese Medicine Syndrome, The Second Affiliated Hospital, Guangzhou University of Chinese Medicine, Guangzhou, PR China*

*^5^ Guangdong Provincial Key Laboratory of New Drug Development and Research of Chinese Medicine, Mathematical Engineering Academy of Chinese Medicine, Guangzhou University of Chinese Medicine, Guangzhou, China*

*^6^ School of Biomedical Sciences, Faculty of Medicine, The Chinese University of Hong Kong, Shatin, N.T., Hong Kong SAR, China*

*** ***Correspondence to:*** *Professor Zhi-Xiu Lin, School of Chinese Medicine, Faculty of Medicine, The Chinese University of Hong Kong, Shatin, N.T., Hong Kong SAR, China. Phone: +852 3943 6347; Fax:* *+852 3942 0941; E-mail: linzx@cuhk.edu.hk*

**Keywords: Pancreatic cancer, Brucein D, Apoptosis, PI3K/Akt, ROS**

**SUPPLEMENTARY METHODS**

**Lentiviral vector transduction**

Capan-2 cells were seeded in 24-well plates at a density of 5×10^4^ cells/well in DMEM media under the above-mentioned culture conditions, and incubated for 14-20 h. The cells were infected by replacing the medium with virus stock (25 μL, virus titer = 1.98×10^8^ TU/mL, the optimal transduction efficiency was MOI = 100) in culture media containing 5 μg/mL of Polybrene, followed by incubation at 37 ˚C for 24 h. After adsorption, the viral solutions were removed, and fresh medium (500 μL) was supplemented. At 72 h post infection, the medium was replaced with 500 μL fresh medium containing 2 μg/mL puromycin for the selection of cells simultaneously carrying EGFP and Luc. The selection was allowed to proceed for 14 d, with medium exchanged every 2 d. The completion of the selection procedure was confirmed as all the un-transduced cells were detached from the well.

**SUPPLEMENTARY FIGURES**

**Figure S1.** BD-elicited apoptosis of PANC-1 and Capan-2 cells was analyzed by the Annexin V-FITC/PI double staining. Cells were treated with 5 µg/mL BD, stained with Annexin V-FITC (green) and PI (red), and measured by the confocal microscopy. DIC: the images obtained by differential interference contrast microscopy; Merge: the merged images of Annexin V-FITC, PI and DIC images.


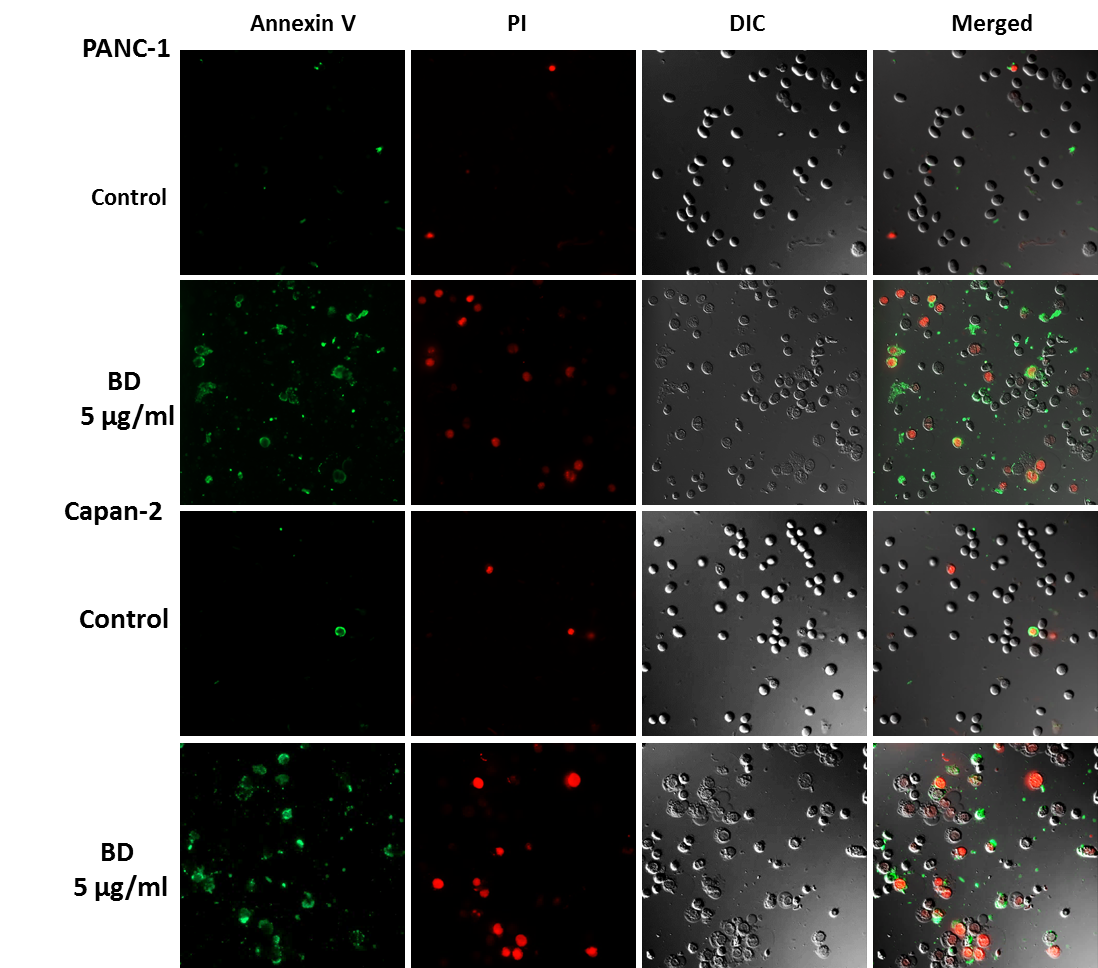


**Figure S2.** The histograms depicted the quantified protein expression (PARP-1, pro-caspase-3, pro-caspase-8, pro-caspase-9, Survivin, XIAP, Bcl-2, Bcl-xL, Bak, Bax and cytosolic cytochrome C), which were normalized by β-actin and presented as fold of control (mean ± SD of three independent experiments), * P < 0.05 and ** P < 0.01 vs the control group.


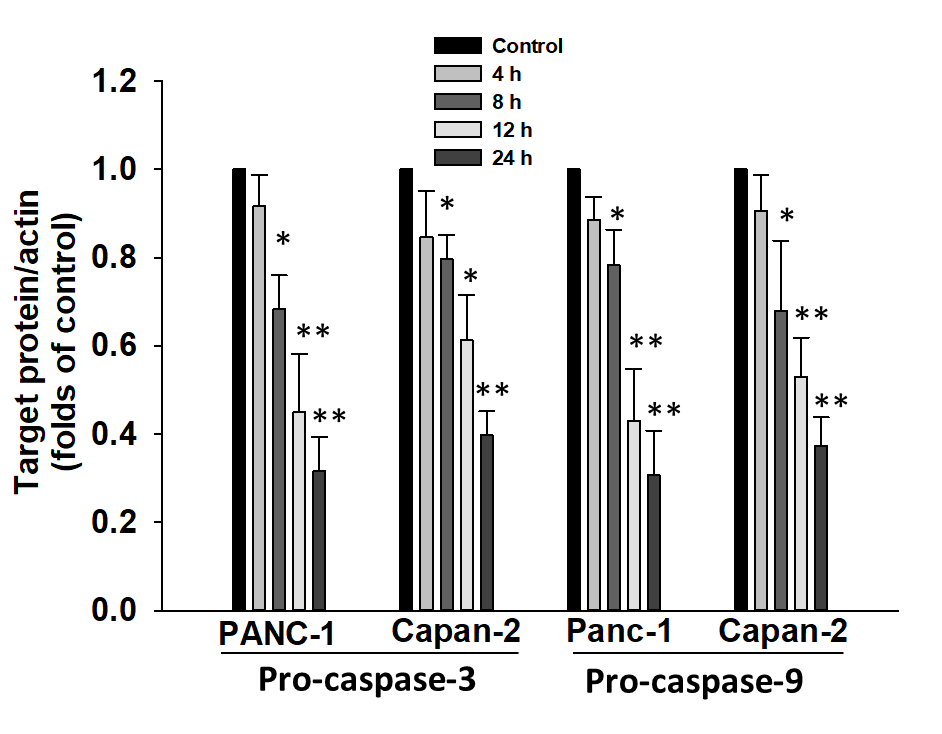

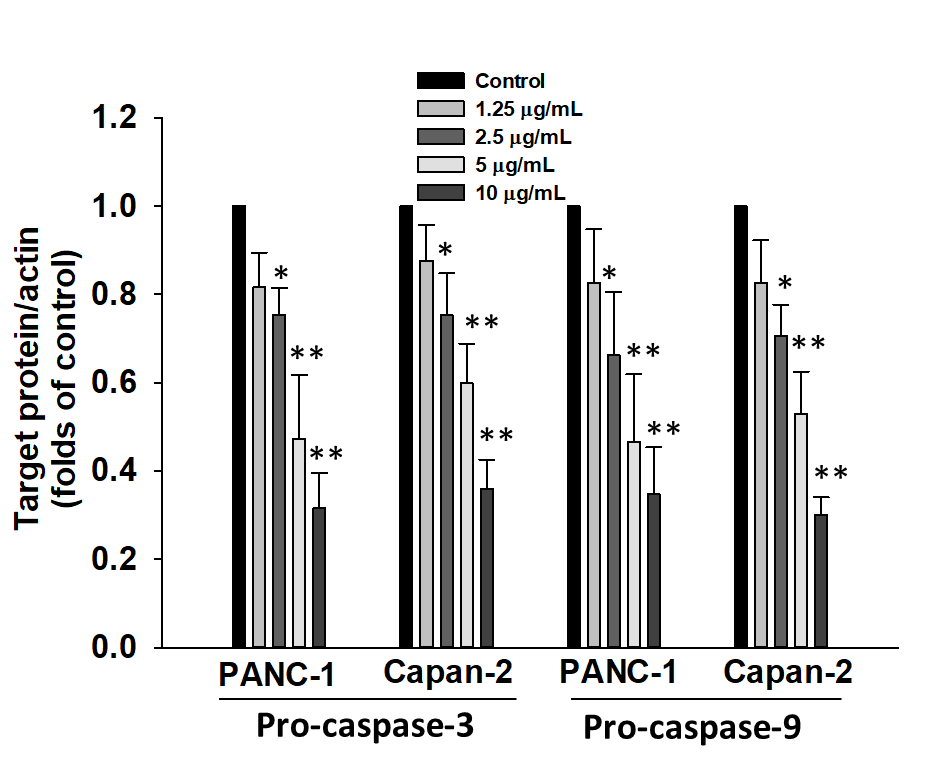


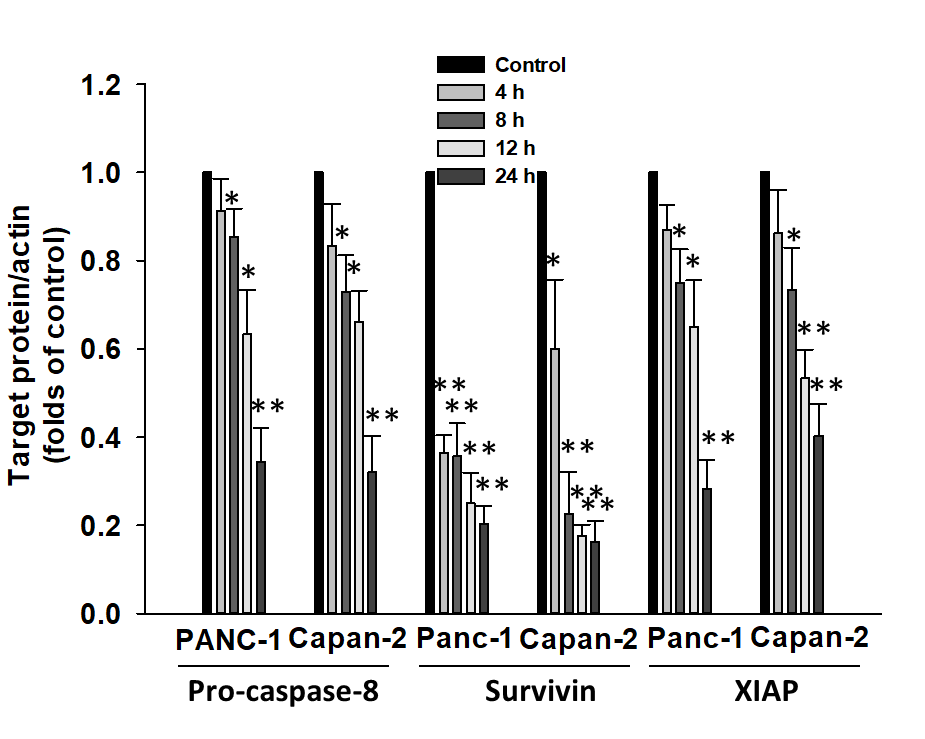

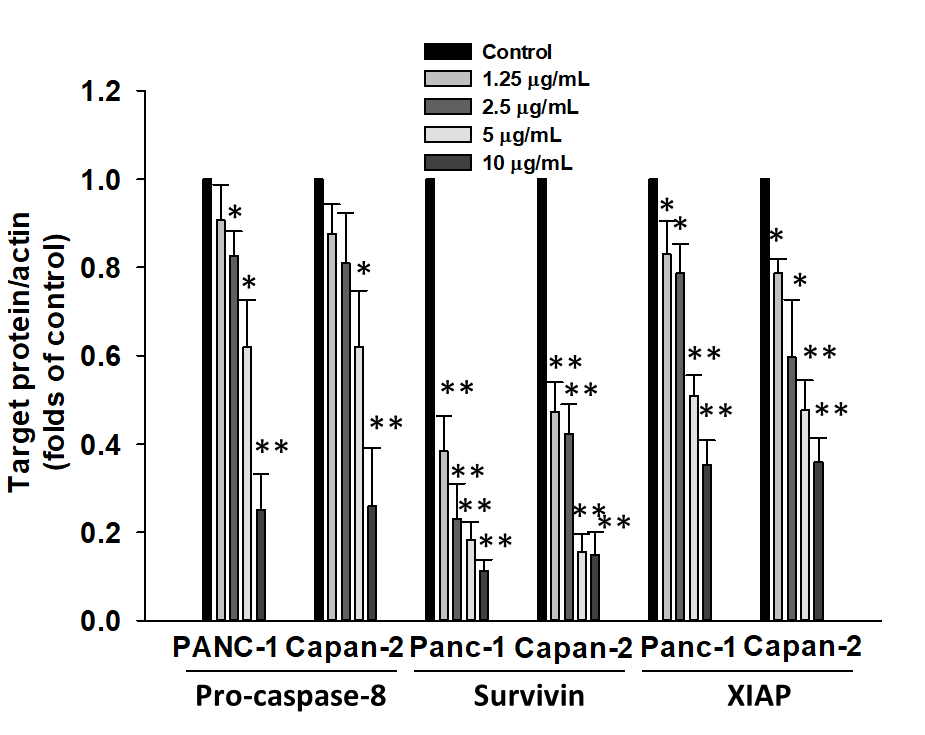


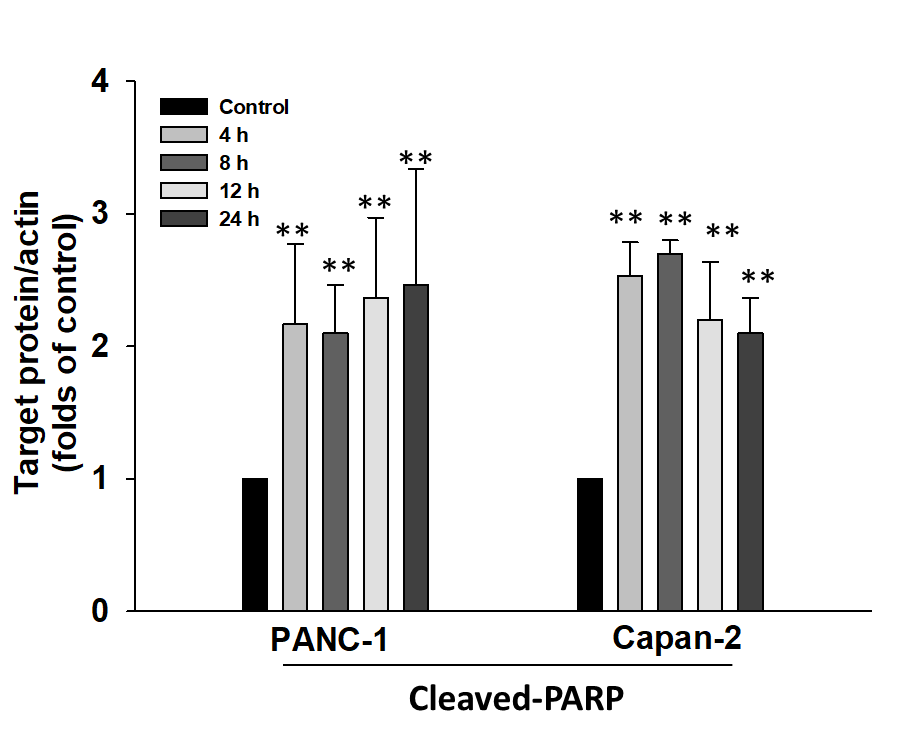

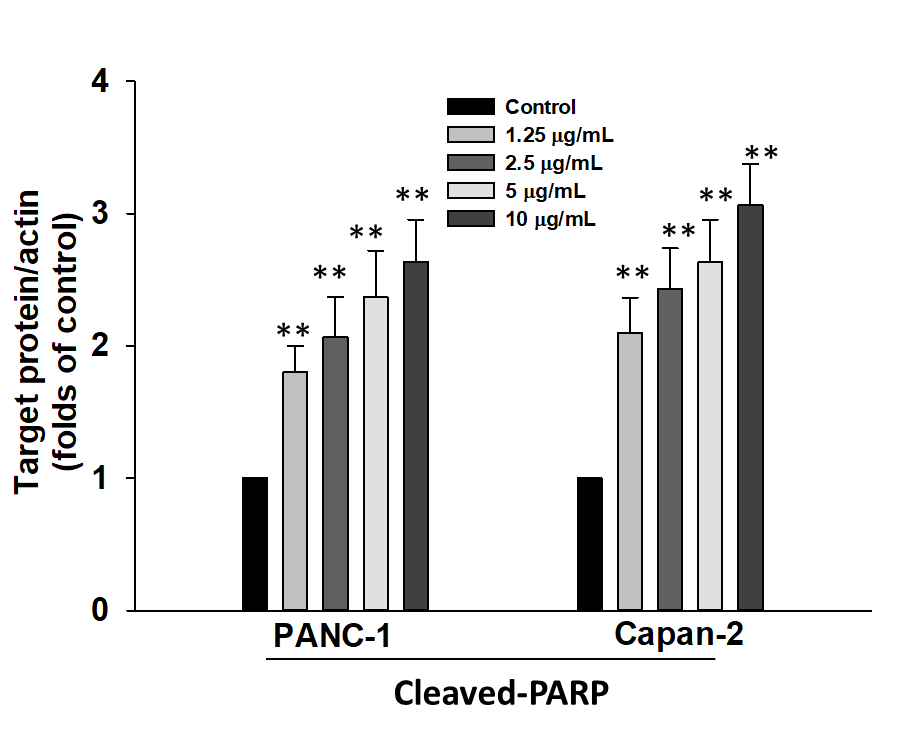


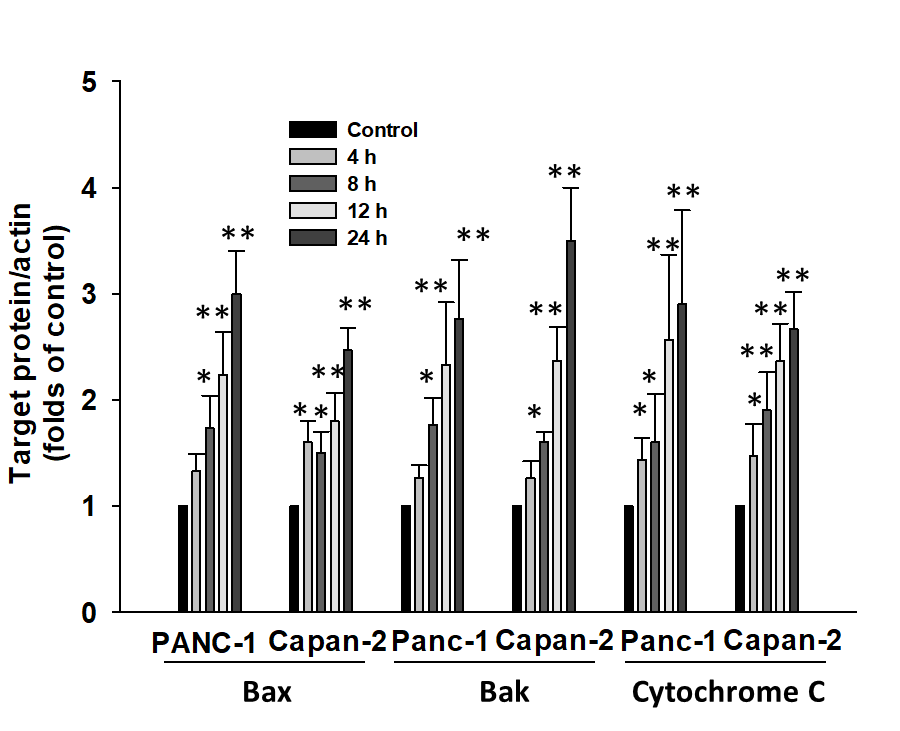

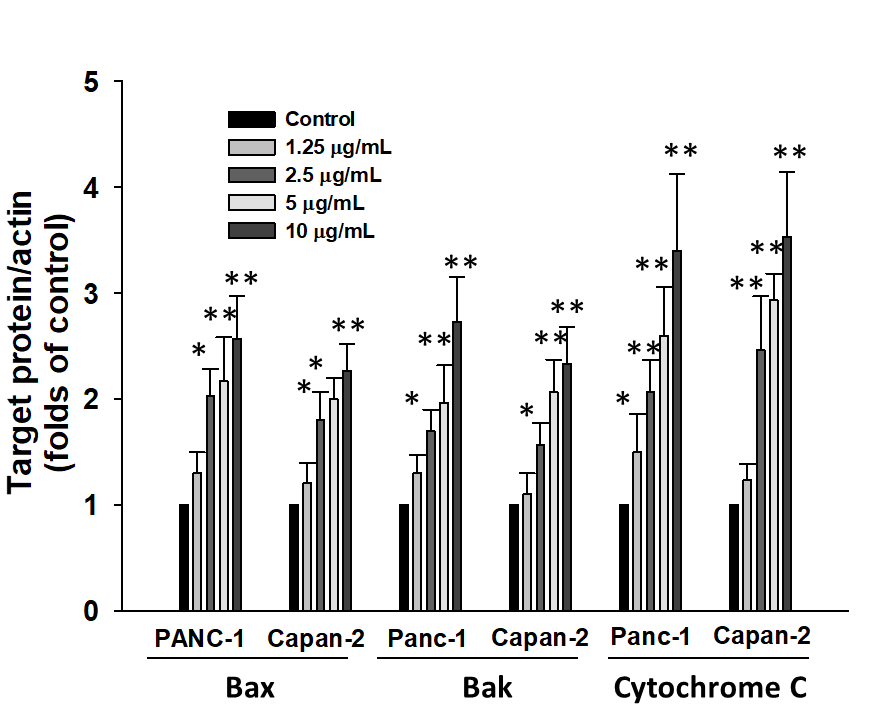


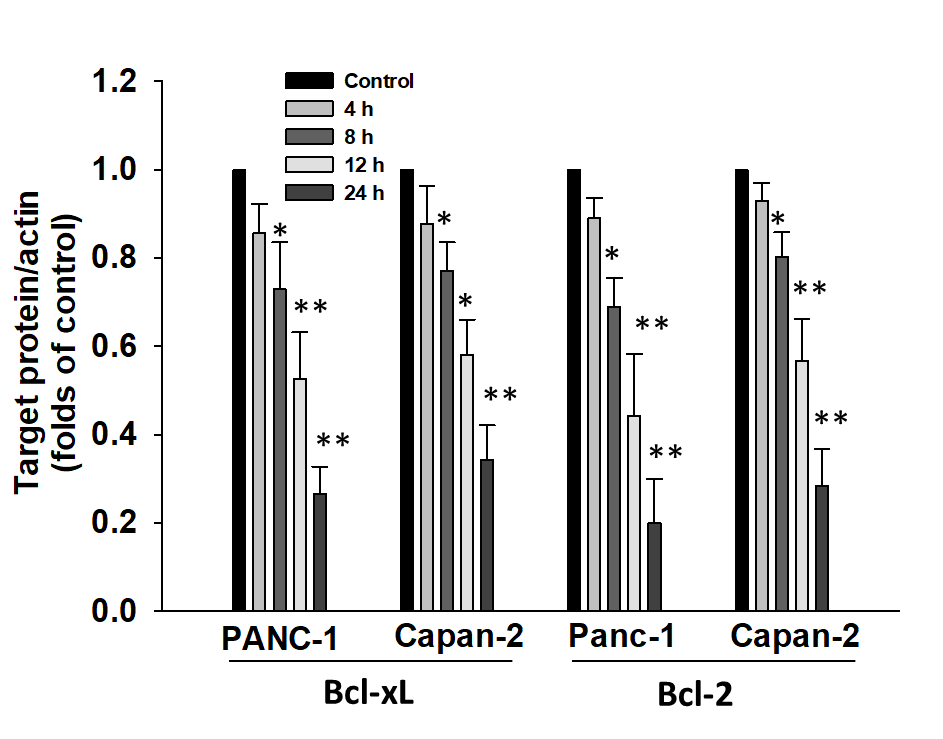

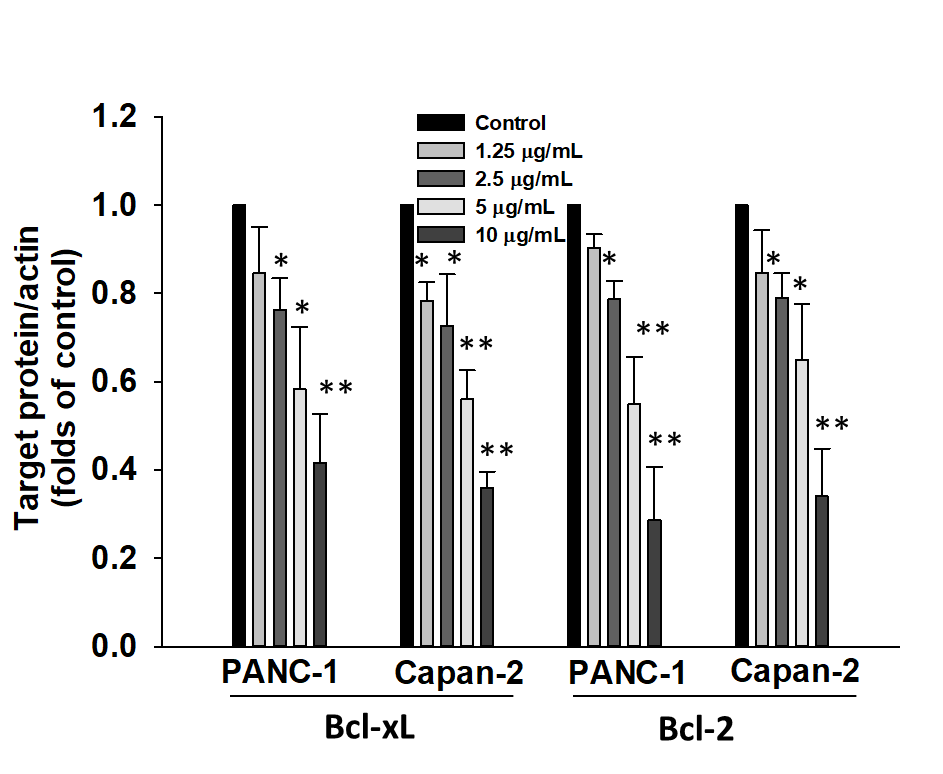


**Figure S3.** The histograms depicted the quantified protein expression (PI3K/Akt and MAPK pathways-related proteins), which were normalized by β-actin and presented as fold of control (mean ± SD of three independent experiments), * P < 0.05 and ** P < 0.01 vs the control group.


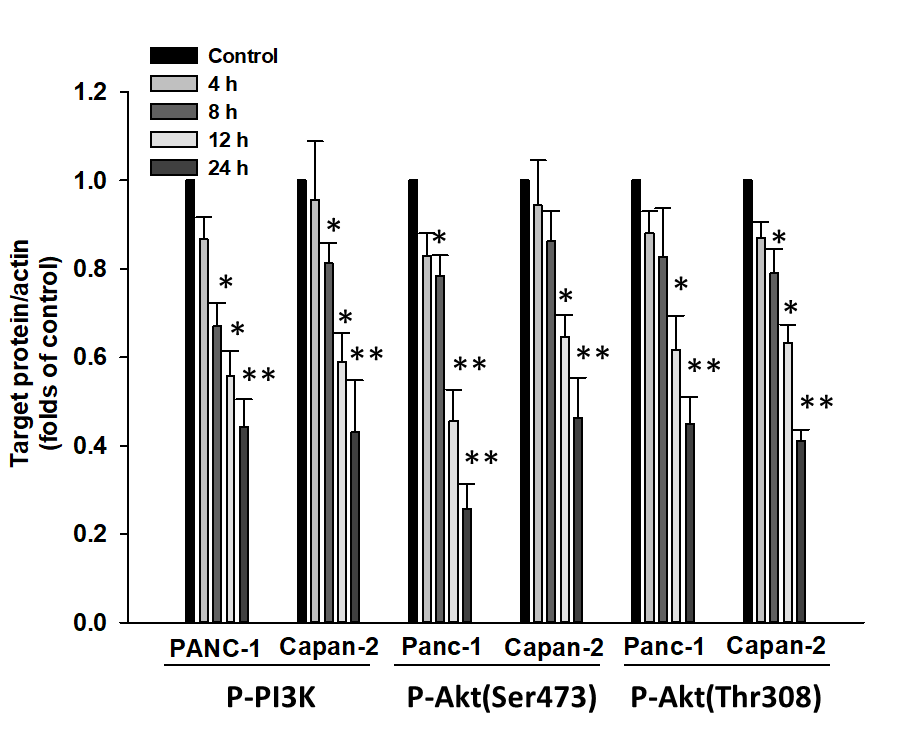

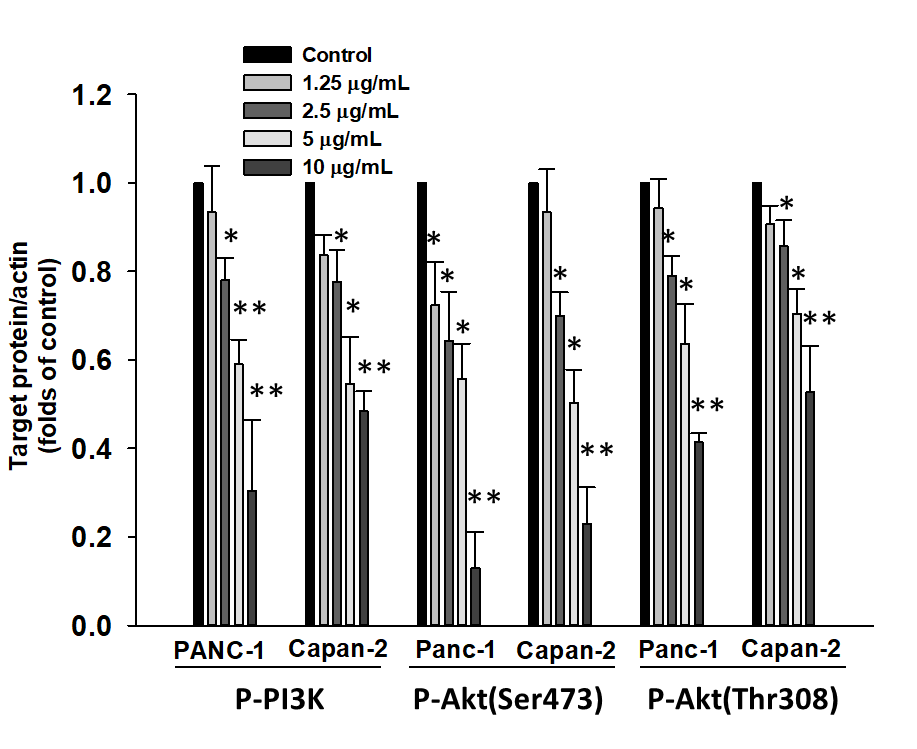


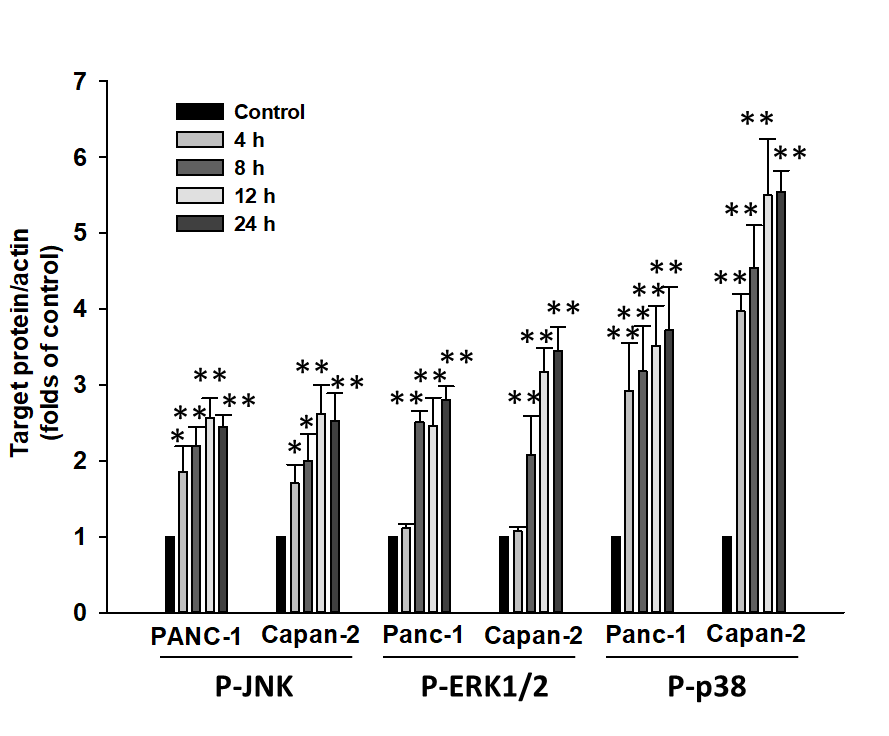

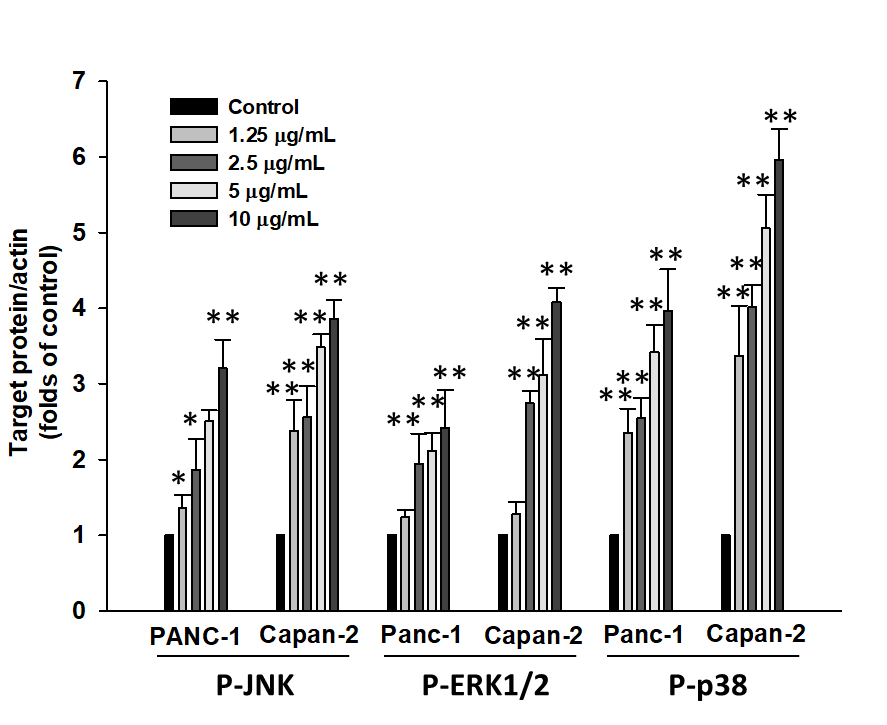


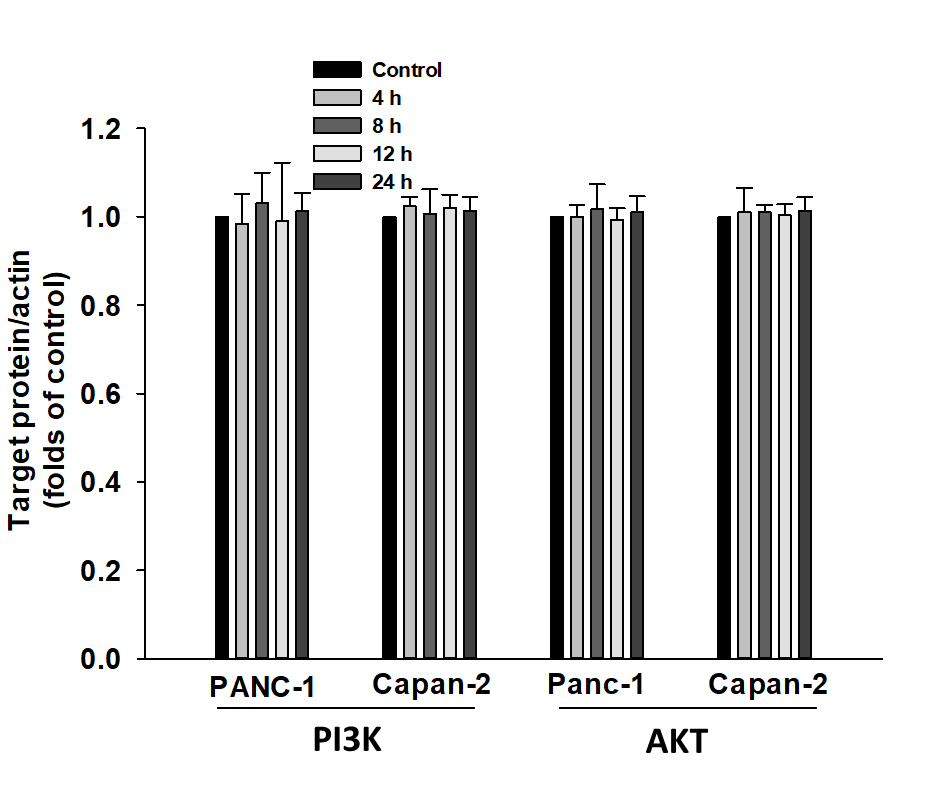

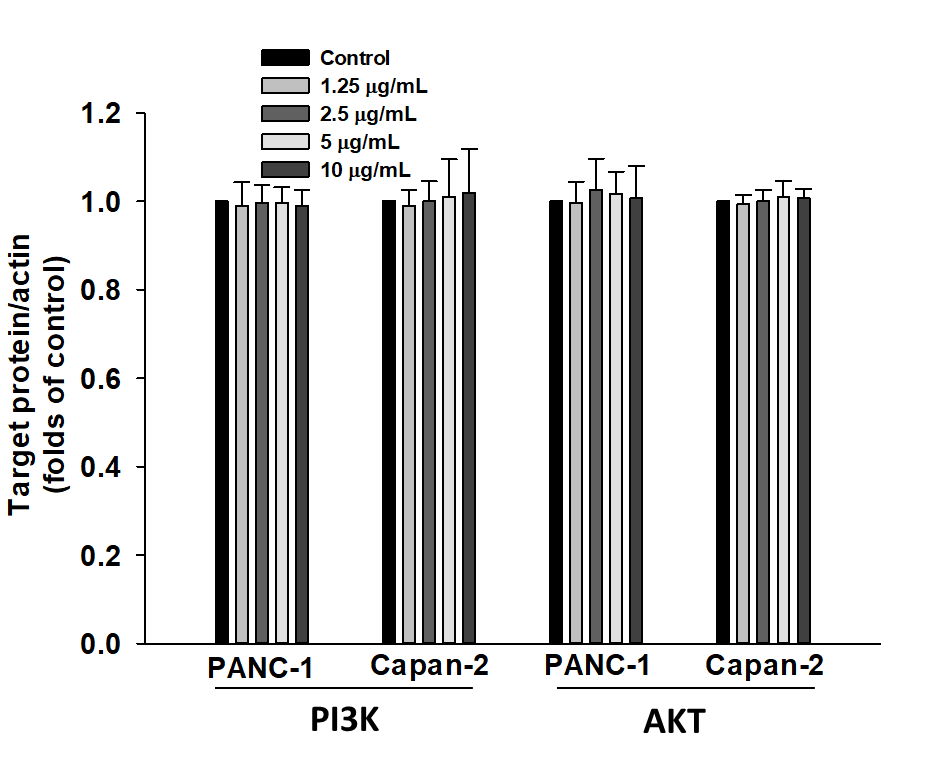


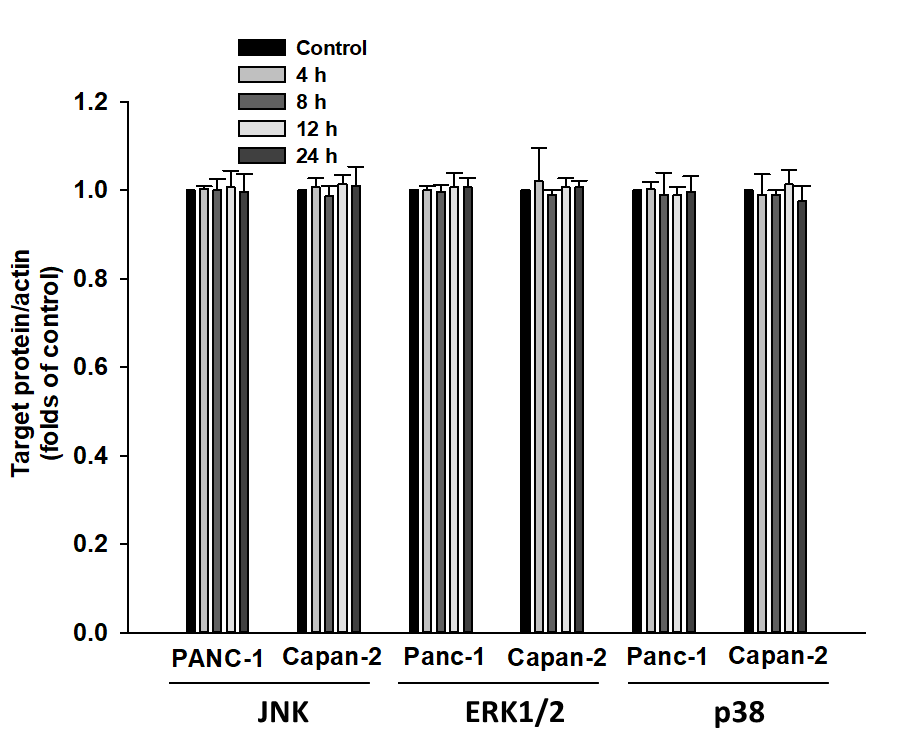

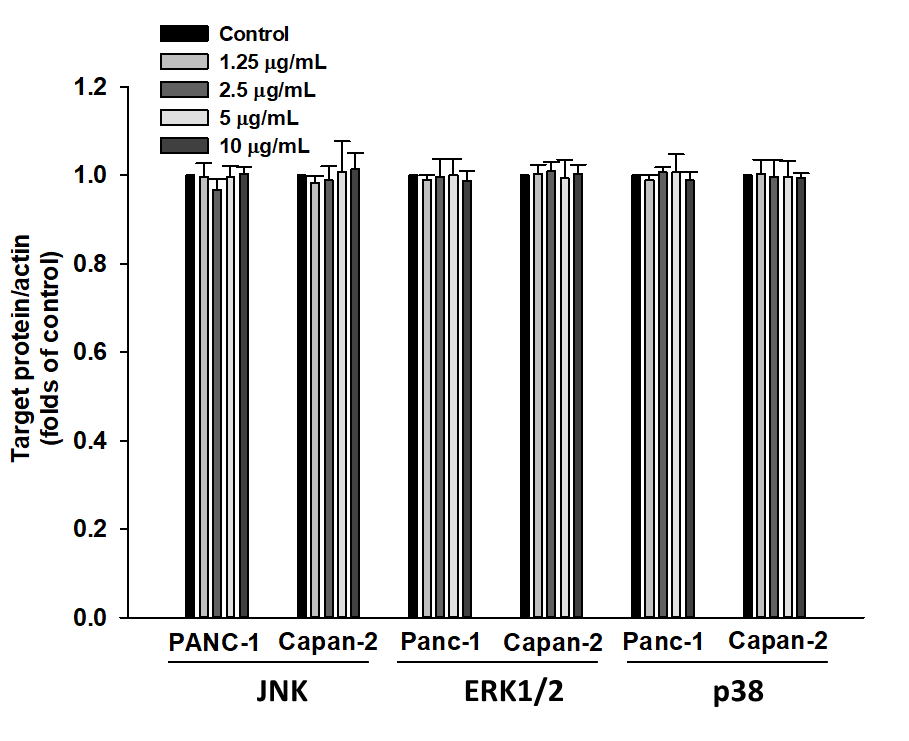


**Figure S4.** Generation of stable EGFP-Luc-PANC-1 and EGFP-Luc-Capan-2 cells. **(A)** Cells were selected based on puromycin resistance after 14 d and EGFP expression was analyzed by confocal laser scanning microscope. Scale bar: 20 μm. Blank: un-transduced Capan-2 cells. **(B)** Transfection efficiency of Lentiviral vector (Plent-CWV-EGFP-linker-Luc-PGK-Puro) detected by flow cytometry in Capan-2 cells. **(C)** Measurement of Luc activity by the Dual-Luc Reporter Assay System. Data presented are the mean ± SD of three independent experiments.


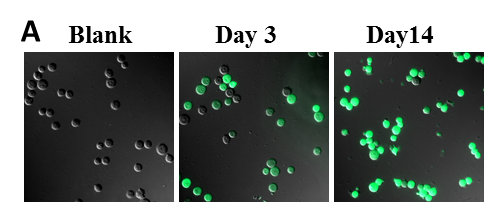


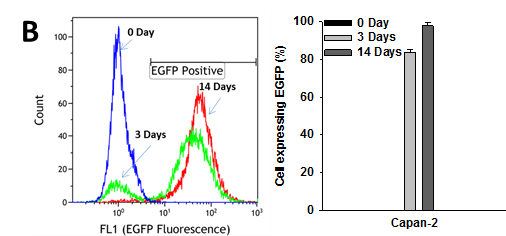


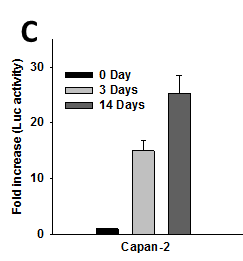


**Figure S5.** Detection of PanCa metastasis by fluorescent imaging. **(A)** *Ex vivo* images of mice subjected to different treatment regimens (a. untreated control; b GEM; c. 5-FU; d. BD1; e. BD2). On 35 d animals were sacrificed and tumor fluorescence was analyzed *ex vivo***. (B)** *Ex vivo* images of mice tissues. Green fluorescence, white light and color images of the vital organs were collected from orthotopic mouse model on the 35th d after treatment.


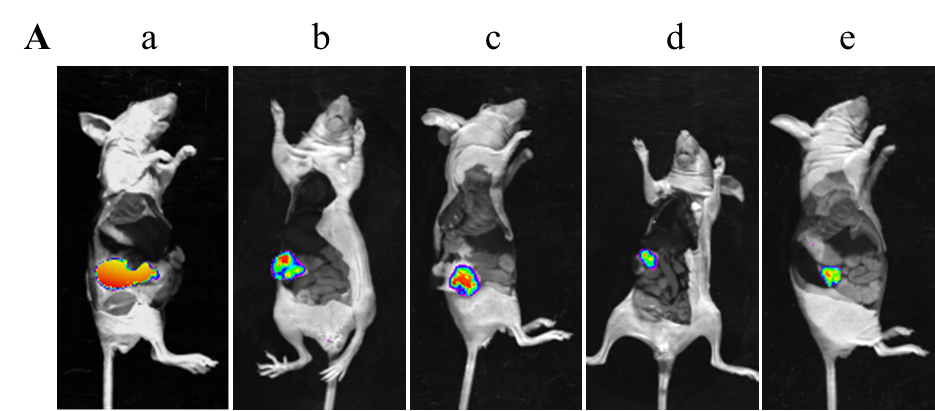


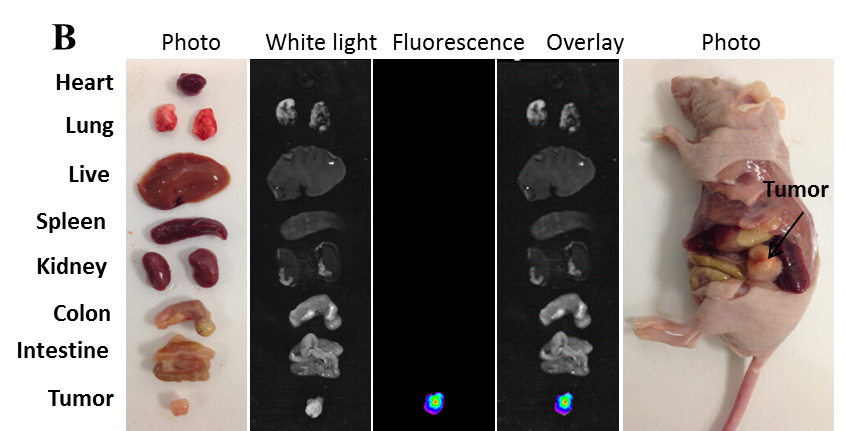


**Figure S6.** Detection of PCNA **(A)** and Ki-67 **(B)** expression by immunofluorescence in pancreatic tumor tissues. Red fluorescence depicted the nuclear expression of PCNA and Ki-67, and blue fluorescence represented cell nuclei with DAPI (4,6-diamidino-2-phenylindole) staining. Images were obtained by a confocal microscope (Zeiss). Scale bars: 20 μm.


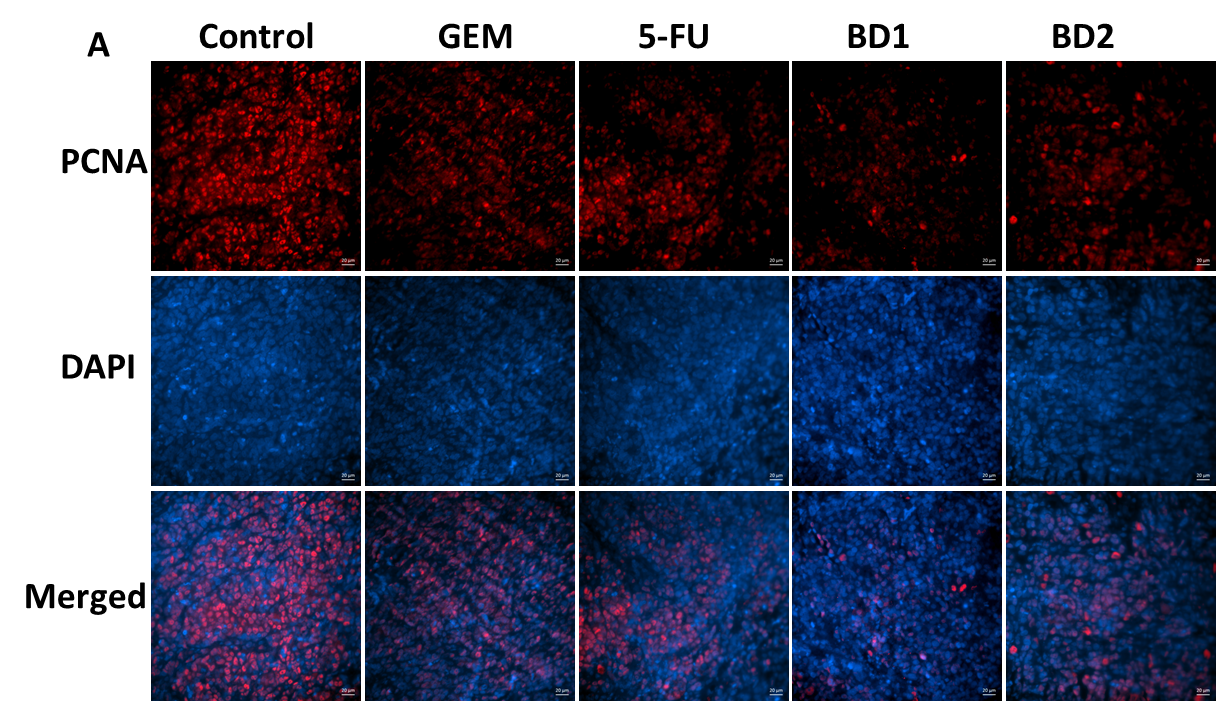


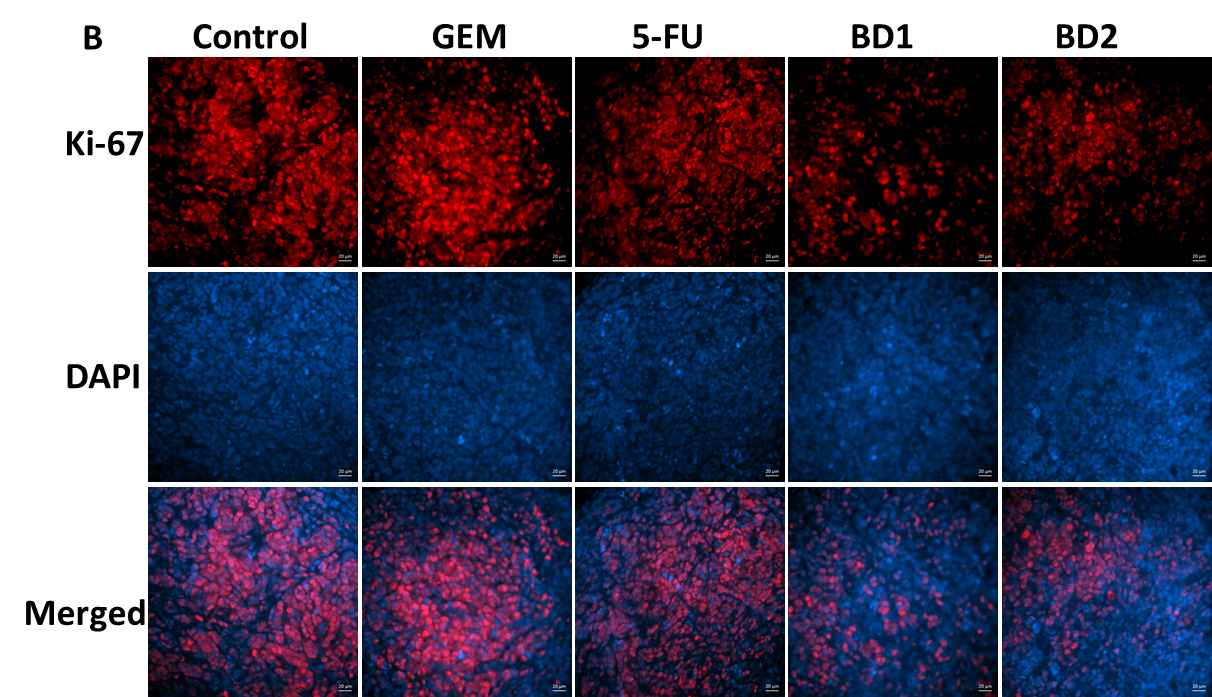


**SUPPLEMENTARY TABLES**

**Table S1.** IC50 values of BD/GEM/5-FU on the growth of PanCa and GES-1 cell lines.

| Cell lines | IC50 (μg/mL) | | | IC50 (μM) | | |
| --- | --- | --- | --- | --- | --- | --- |
|  | GEM | 5-FU | BD | GEM | 5-FU | BD |
| Capan-1 | 1.75 | 2.24 | 0.80 | 6.65 | 17.23 | 1.95 |
| Capan-2 | >200 | 2.03 | 1.15 | >200 | 15.57 | 2.80 |
| PANC-1 | 22.00 | 9.02 | 3.01 | 83.6 | 69.33 | 7.33 |
| SW-1990 | 28.37 | 5.03 | 10.30 | 107.81 | 38.64 | 25.09 |
| GES-1 | 0.13 | 1.66 | >200 | 0.48 | 12.74 | >200 |

**Table S2.** Biochemical parameters from nude mice on 35 d for toxicity evaluation.

| Treatment | ALT (U/L) | AST (U/L) | LDH (U/L) | CK (U/L) | Creatinine (mg/dL) |
| --- | --- | --- | --- | --- | --- |
| Control | 16.95±2.35 | 37.34±3.37 | 1373.85±420.58 | 108.03±18.56 | 2.6±0.57 |
| GEM | 19.56±2.91 | 40.55±3.16 | 1541.93±386.50 | 101.74±5.51 | 2.6±0.41 |
| 5-FU | 16.97±2.66 | 34.82±4.73 | 1601.08±300.35 | 118.05±43.7 | 2.55±0.19 |
| BD1 | 16.07±1.40 | 34.73±1.97 | 1547.67±396.08 | 104.61±37.55 | 3.21±0.45 |
| BD2 | 15.55±2.82 | 35.84±2.31 | 1449.19±276.89 | 113.85±8.16 | 3.37±0.39 |

Notes: Data are expressed as the mean ± SD. AST: Aspartate Transaminase; ALT: Alanine Transaminase; LDH: Lactate Dehydrogenase; CK: Creatine Kinase. There was no significant variation among biochemical parameters of mice between control and treatment groups (P > 0.05).
